# Upregulation of HBV transcription by sodium taurocholate cotransporting polypeptide at the postentry step is inhibited by the entry inhibitor Myrcludex B

**DOI:** 10.1038/s41426-018-0189-8

**Published:** 2018-11-21

**Authors:** Kaitao Zhao, Shuhui Liu, Yingshan Chen, Yongxuan Yao, Ming Zhou, Yifei Yuan, Yun Wang, Rongjuan Pei, Jizheng Chen, Xue Hu, Yuan Zhou, He Zhao, Mengji Lu, Chunchen Wu, Xinwen Chen

**Affiliations:** 1 State Key Laboratory of Virology, Wuhan Institute of Virology, Chinese Academy of Sciences, 430071 Wuhan, China; 2 University of Chinese Academy of Sciences, 100049 Beijing, China; 3 Shenzhen Xenotransplantation Research and Development Center, State and Local Joint Cancer Genome Clinical Application of Key Technology Laboratory, Shenzhen Second People’s Hospital, First Affiliated Hospital of Shenzhen University, 518035 Shenzhen, China; 4 Institute of Virology, University Hospital of Essen, 45147 Essen, Germany

## Abstract

Sodium taurocholate cotransporting polypeptide (NTCP) is a functional receptor for hepatitis B virus (HBV) entry. However, little is known regarding whether NTCP is involved in regulating the postentry steps of the HBV life cycle. Here, we found that NTCP expression upregulated HBV transcription at the postentry step and that the NTCP-targeting entry inhibitor Myrcludex B (MyrB) effectively suppressed HBV transcription both in an HBV in vitro infection system and in mice hydrodynamically injected with an HBV expression plasmid. Mechanistically, NTCP upregulated HBV transcription via farnesoid X receptor α (FxRα)-mediated activation of the HBV EN2/core promoter at the postentry step in a manner that was dependent on the bile acid (BA)-transport function of NTCP, which was blocked by MyrB. Our findings uncover a novel role for NTCP in the HBV life cycle and provide a reference for the use of novel NTCP-targeting entry inhibitors to suppress HBV infection and replication.

## Introduction

With ~240 million chronically infected individuals worldwide, Hepatitis B virus (HBV) infection remains a major global health burden^1^. Persistent HBV infection leads to liver cirrhosis and hepatocellular carcinoma^2^. Although currently approved clinical treatments for HBV infection based on interferon alpha (IFN-α) or nucleos(t)ide analogs provide reasonable control of viral production in chronic infection, HBV clearance and subsequent seroconversion only occur in a minority of patients. Overall, HBV eradication and/or resolution of infection remains a challenge^3^. Therefore, the development of new anti-HBV agents targeting other steps of the HBV life cycle is urgently needed.

As sodium taurocholate cotransporting polypeptide (NTCP) has been identified as the functional receptor for HBV entry^4^, entry inhibitors targeting NTCP offer a promising novel therapeutic option^5^. One such entry inhibitor is Myrcludex B (MyrB), a myristoylated synthetic lipopeptide comprising 47 amino acids derived from the preS1 domain of the HBV large surface protein. MyrB can efficiently block de novo HBV and HDV infection both in vitro^6^ and in vivo^7^. Moreover, MyrB administration may also prevent intrahepatic viral spread in HBV-infected uPA/SCID mice^8^. MyrB was recently confirmed to be well tolerated in healthy volunteers^9^, with antiviral efficacy against HBV and HDV infection^10^.

In addition to being the receptor for HBV and HDV, NTCP is an important bile salt transporter. NTCP is responsible for the hepatic uptake of ~80% of conjugated bile acids (BAs) from the blood^11^. BAs activate a range of dedicated nuclear receptors (NRs), such as the nuclear receptor farnesoid X receptor α (FxRα); these NRs play crucial roles in the transcriptional control of critical steps of certain hepatic functions ranging from BAs homeostasis to hepatic lipids and glucose metabolism^12,13^. Additionally, BAs promote HBV transcription and gene expression, which are mediated by FxRα^13^. A recent study suggests that the overexpression of NTCP might contribute to HBV protein expression and DNA replication at the postentry step^14^, although the mechanism remains unclear.

In this study, we investigated the impact of NTCP on aspects of HBV replication other than HBV entry. Our results showed that NTCP expression upregulated HBV replication at the transcriptional level, and MyrB counteracted this NTCP-mediated upregulation of HBV replication at the postentry step both in vitro and in vivo. Subsequent mechanistic analysis revealed that the upregulation of HBV transcription by NTCP was mediated by its bile acid (BA)-transporter function via FxRα-mediated activation of the HBV EN2/core promoter. These findings deepen our understanding of the role of NTCP, independent of its receptor function, in the HBV life cycle. Moreover, the findings indicate that MyrB may possess antiviral activity independent of its entry-suppressing effect, and this report will provide a reference for the use of novel entry inhibitors as therapeutic strategies to interfere with HBV infection and replication.

## Results

### NTCP expression upregulated HBV replication at the postentry step

**Fig. 1:**
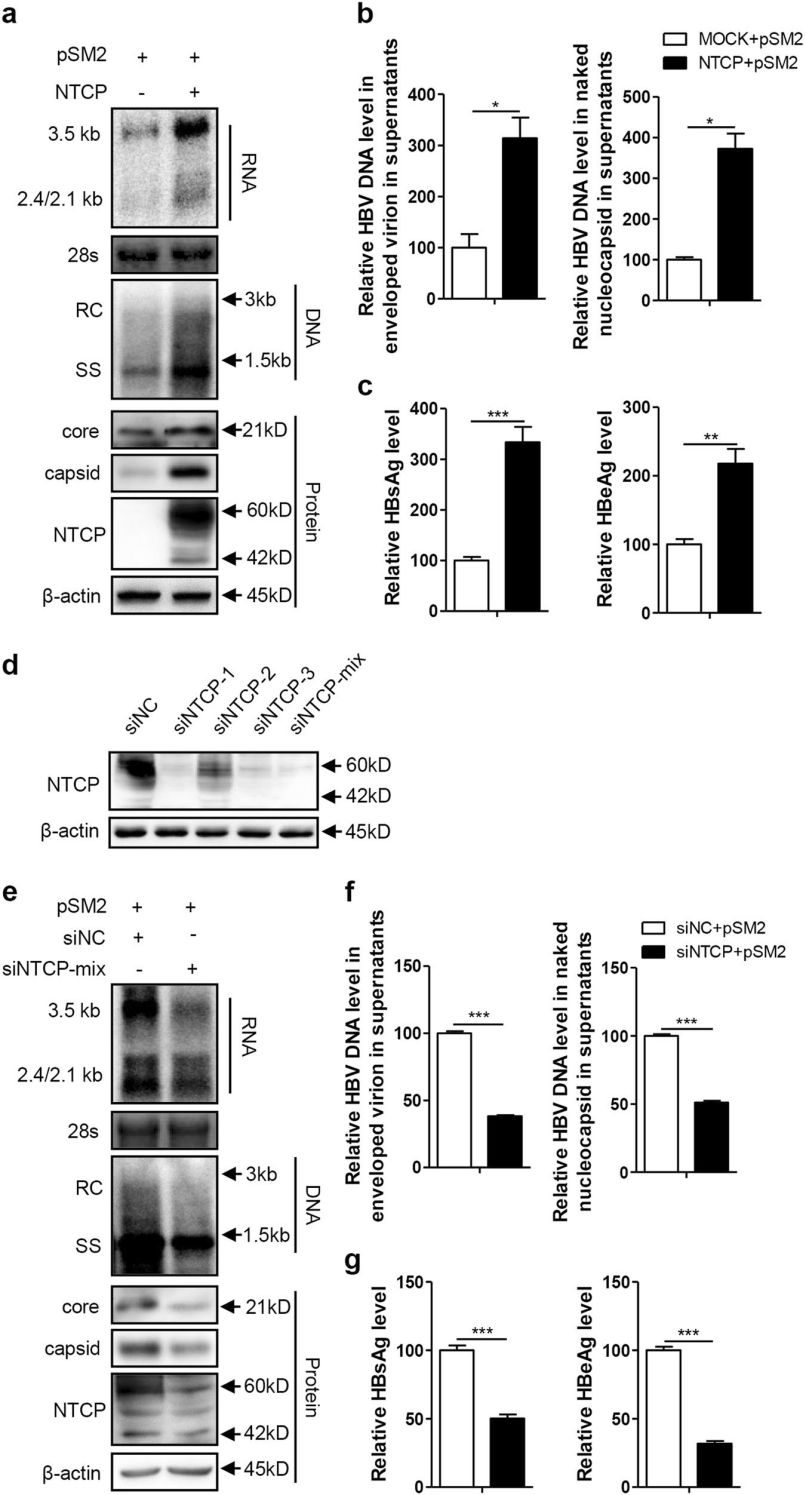
NTCP upregulates HBV replication at the postentry step. Huh7 and Huh7-NTCP cells transfected with the indicated plasmids or siRNAs were harvested at 72 h post transfection (hpt). **a**, **e** HBV transcription and replication intermediates were detected by northern blotting and Southern blotting, respectively (top panel). The positions of relaxed circular (RC) and single-stranded (SS) DNAs and HBV 3.5, 2.4, and 2.1-kb RNAs are indicated. The 28S ribosomal RNA served as a loading control. Expression of NTCP and core proteins and formation of capsids in the cells were analyzed by western blotting (bottom panel). Two bands of NTCP proteins, 60-kDa glycosylated and 42-kDa nonglycosylated, are indicated. β-actin served as a loading control. **b**, **f** Enveloped virions and naked nucleocapsids in culture supernatants were immunoprecipitated with anti-HBsAg and anti-core antibodies, respectively. HBV DNA was detected by real-time PCR (*n* = 3). **c**, **g** HBsAg and HBeAg in culture supernatants were detected by enzyme-linked immunosorbent assay (ELISA) (*n* ≥ 4). **d** The knockdown effects of the different siRNAs targeting NTCP were detected by western blotting. A two-tailed *t*-test was used to determine differences in multiple comparisons. *P < 0.05, **P < 0.01, ***P < 0.001

The Huh7-NTCP cell line stably expressing human NTCP (hNTCP) was transfected with the replication-competent HBV plasmid pSM2 to investigate whether NTCP affects other aspects of HBV replication, in addition to viral entry. As shown in Fig. 1a, the NTCP expression in the Huh7 cells significantly increased the levels of intracellular HBV RNA and DNA. Correspondingly, the amount of HBV DNA secreted into cell culture supernatants as two major types of viral particles—enveloped virions and naked nucleocapsids—was significantly increased by approximately 3.1-fold and 3.5-fold, respectively (Fig. 1b). The levels of HBV core protein and capsid in the cell lysates were also increased (Fig. 1a). Moreover, the levels of HBsAg and HBeAg in the culture supernatants were also increased by approximately 3.3-fold and 2.2-fold, respectively, in the presence of the NTCP (Fig. 1c). These results suggest that NTCP expression may facilitate HBV replication at the postentry step. To further validate these results, we employed a specific small interfering RNA (siRNA) mixture to silence NTCP expression in the Huh7-NTCP cells, and the effect of the NTCP silencing was verified (Fig. 1d), with a significant reduction in HBV RNA and HBV DNA observed in both the cell lysates and supernatants (Fig. 1e, f). The levels of HBV core protein and capsid in the cell lysates were also reduced (Fig. 1e). Consistently, HBsAg and HBeAg decreased by 50% and 68%, respectively, in the culture supernatants (Fig. 1g). The impact of NTCP expression on replication of the different HBV genotypes was further assessed, demonstrating that replication of the HBV A-C genotypes was enhanced by the NTCP, though at different efficiencies (Fig. S1).

Previous studies have shown that although it retains the function of BA transport, hNTCP with amino acids (aa) 157–165 replaced by the corresponding monkey sequence (mkNTCP) does not support HBV entry^15^. To rule out the possibility that newly produced HBV virions from pSM2-transfected cells infect Huh7-NTCP cells, we introduced a mutated protein, NTCP/mk, consisting of hNTCP with aa 157–165 having been replaced by the mkNTCP sequence. We found that the NTCP/mk was still able to increase the HBV RNA, DNA, and HBsAg levels, albeit at a reduced rate compared with that of the wild-type NTCP (Fig. S2).

Furthermore, we investigated whether the NTCP can upregulate HBV replication in a dose-dependent manner. A gradient concentration of pcDNA3.1-NTCP plasmids was cotransfected with the HBV replication-competent plasmid pSM2. Accordingly, dose-dependent increases in intracellular HBV RNA, DNA, core protein, and capsid were observed (Fig. S3a); dose-dependent decreases were also observed for the HBV DNA levels contained in the secreted enveloped virions and naked nucleocapsids, as well as in the secreted HBsAg (Fig. S3b, c).

Taken together, these results indicate that NTCP expression significantly enhanced HBV replication at the postentry step.

### Entry inhibitor Myrcludex B (MyrB) abolished the NTCP-mediated upregulation of HBV replication

**Fig. 2:**
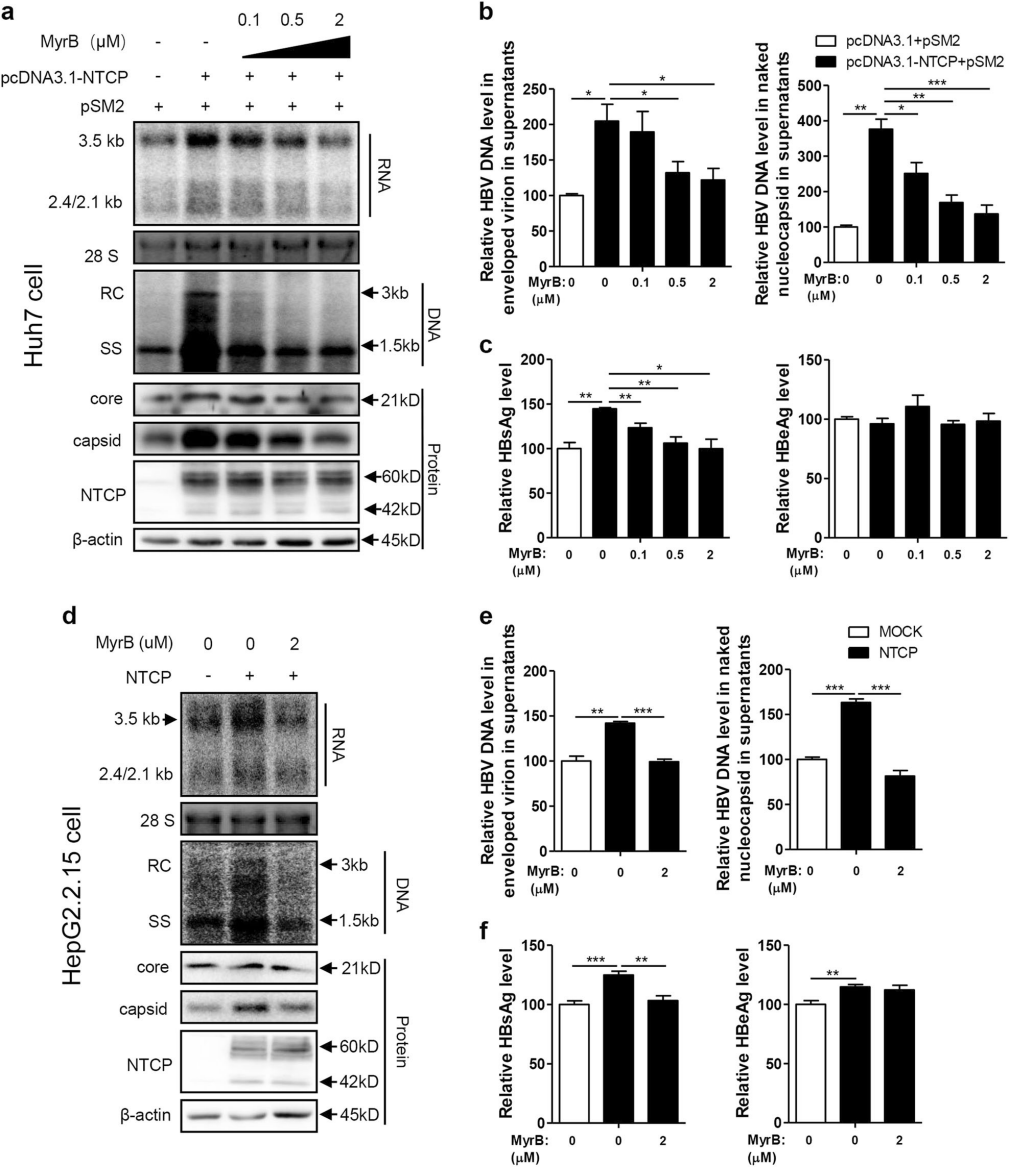
MyrB inhibits HBV replication in NTCP-expressing cells. Huh7 cells transfected with the indicated plasmids, HepG2.2.15 cells and HepG2.2.15-NTCP cells were treated with MyrB at the indicated concentrations. **a**, **d** HBV transcription and replication intermediates were detected by northern blotting and Southern blotting, respectively (top panel). Expression of NTCP and core proteins and capsid formation in cells were analyzed by western blotting (bottom panel). **b**, **e** HBV DNA in enveloped virions and naked nucleocapsids in culture supernatants were detected by real-time PCR (*n* ≥ 3). **c**, **f** HBsAg and HBeAg in the culture supernatants were detected by ELISA (*n* ≥ 4). A two-tailed *t*-test was used to determine differences in the multiple comparisons. *P < 0.05, **P < 0.01, ***P < 0.001

The NTCP-targeting entry inhibitor MyrB is a promising novel candidate against HBV and is currently in clinical development^16^. Therefore, the impact of MyrB on NTCP-mediated upregulation of HBV replication was further investigated. First, the potential cytotoxicity of MyrB treatment on hepatoma cells was ruled out (Fig. S4a). As shown in Fig. 2a, MyrB reduced the NTCP-dependent increases in HBV RNA, DNA, core protein, and capsid levels in a dose-dependent manner, with a marked effect at 2 μM. Correspondingly, MyrB treatment also caused dose-dependent decreases in secreted HBV DNA (Fig. 2b) and HBsAg (Fig. 2c). Consistent results were obtained in experiments using HepG2.2.15 cells with stable NTCP expression (Fig. 2d–f) and HBV A-C genotypes (Fig. S1). These findings indicate that MyrB can abolish the NTCP-mediated upregulation of HBV replication.

### MyrB inhibited HBV replication at the postentry step in an HBV in vitro infection system

**Fig. 3:**
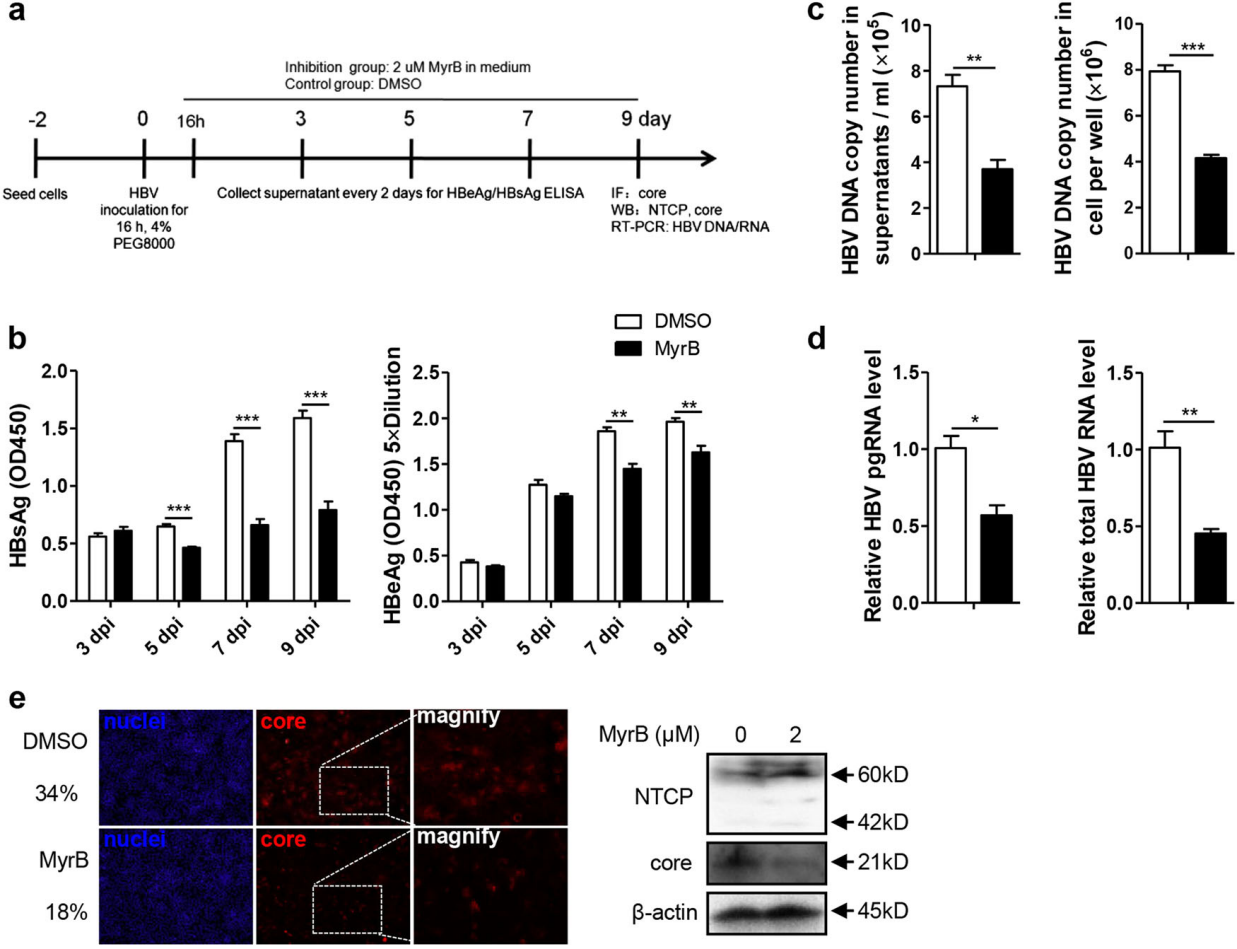
MyrB inhibits HBV replication at the postentry step in an in vitro infection system. **a** Experimental layout: Huh7-NTCP cells were seeded in 12-well plates and inoculated with HBV in the presence of 4% PEG8000 for 16 h. Next, the cells were cultured with culture medium with 2 μM MyrB or equivoluminal DMSO added. **b** HBsAg and HBeAg were analyzed by ELISA (*n* = 4). **c** Supernatant HBV DNA, intracellular HBV DNA, **d** HBV pgRNA and total RNA were detected by real-time PCR (*n* ≥ 3). **e** Cells were fixed and immunostained with an antibody against the core protein (red) (magnification ×100); intracellular core protein was also detected by western blotting. A two-tailed *t*-test was used to determine differences in multiple comparisons. *P < 0.05, **P < 0.01, ***P < 0.001

As hepatoma cell lines with stable NTCP expression have been used for in vitro HBV infection^4,17^, we further investigated the effect of MyrB on HBV at the postentry step using the same in vitro infection system (Fig. 3a), and MyrB was added to the culture medium at 16 h after HBV infection and sustained for 9 days^18^. As shown in Fig. 3b, MyrB significantly reduced the levels of the HBsAg in cell culture supernatants by 29%, 53%, and 50% at 5, 7, and 9 dpi, respectively. HBeAg levels were also reduced by 22% and 17% at 7 and 9 dpi, respectively. In addition, at 9 dpi, both extracellular and intracellular HBV DNA, pgRNA and total RNA levels were decreased by 49%, 48%, 43%, and 55%, respectively (Fig. 3c, d). According to immunofluorescence and western blot assays, the core protein expression level significantly decreased after the MyrB treatment (Fig. 3e). Thus, MyrB inhibited the HBV replication at the postentry step in an HBV in vitro infection system.

### MyrB inhibited HBV replication by targeting mouse NTCP in vivo

**Fig. 4:**
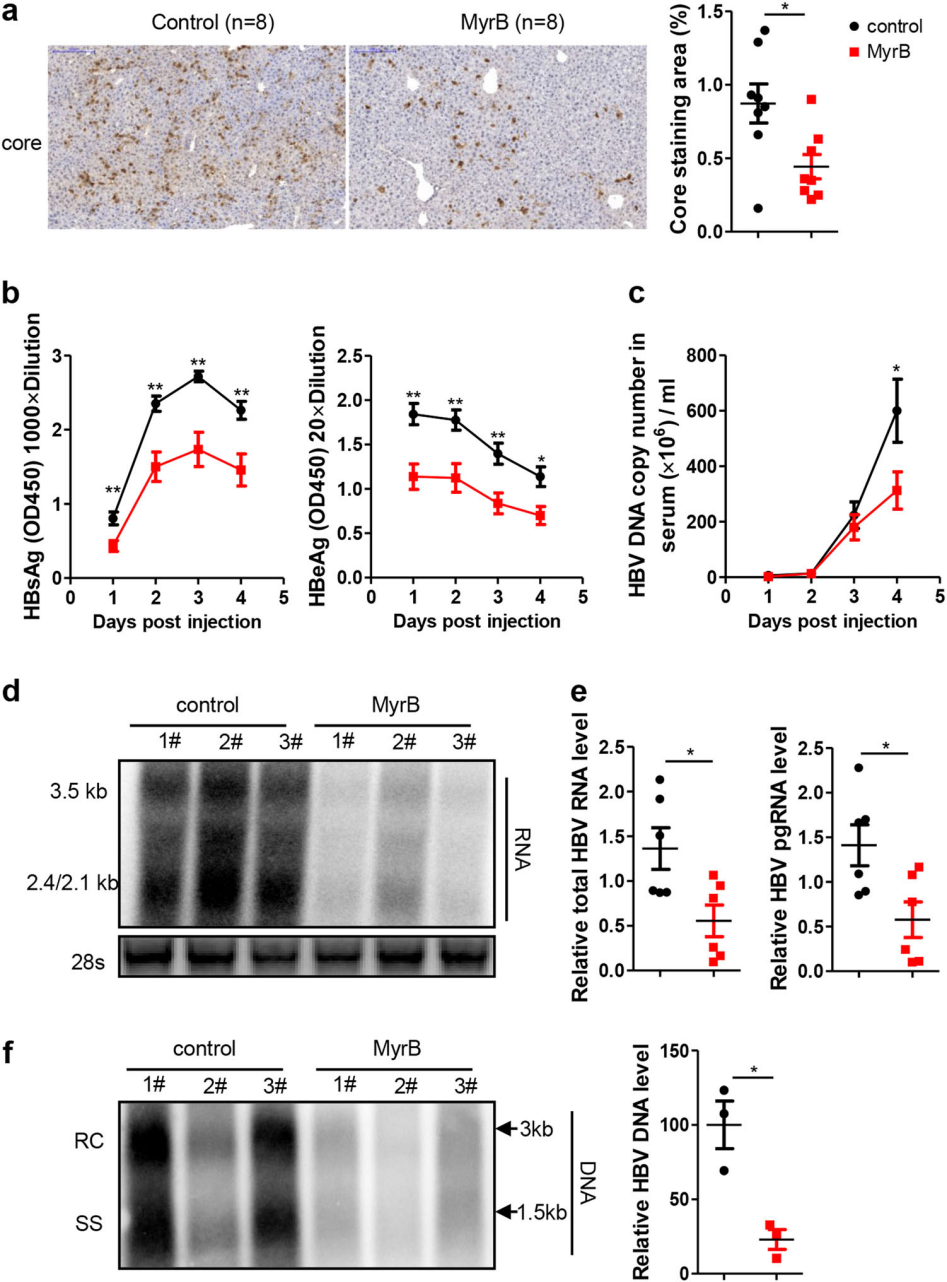
MyrB inhibits HBV replication at the postentry step in vivo. **a** Intrahepatic core proteins were detected by immunohistochemical staining at 4 days after hydrodynamic injection (dphi). The percentage of area showing stained core proteins was analyzed using a Panoramic MIDI Digital Slide Scanner (3DHISTECH). **b** HBsAg and HBeAg secreted in serum were detected by ELISA. **c** HBV DNA in serum was detected by real-time PCR. **d**, **e** HBV RNA in the liver was analyzed by northern blotting and real-time PCR, respectively. mRNA levels were normalized to mouse *GAPDH* (m*GAPDH*) mRNA. **f** HBV DNA in the liver was analyzed by Southern blotting, and the intensity of each lane was calculated using NIH ImageJ software. Data are representative of two independent experiments. A two-tailed *t*-test was used to determine the differences in multiple comparisons. *P < 0.05, **P < 0.01

MyrB can bind to mouse NTCP and block its BA-transporter function^19^; therefore, the antiviral effect of MyrB on HBV was further assessed in mice. Because HBV infection is not supported in mice^20^, we hydrodynamically injected the HBV replication-competent plasmid pSM2 into C57BL/6 mice. Immunohistochemistry (IHC) staining showed obvious suppression of the level of HBV core protein by the MyrB treatment (Fig. 4a). The levels of HBsAg, HBeAg (Fig. 4b) and HBV DNA (Fig. 4c) in the mouse sera were also significantly decreased by MyrB administration. Consistent results were obtained for HBV RNA (Fig. 4d, e) and DNA (Fig. 4f) in the liver. These results confirmed that the MyrB treatment inhibited HBV replication by targeting mouse NTCP in vivo.

### MyrB abolished NTCP-mediated upregulation of HBV replication by inhibiting NTCP-mediated BA uptake

**Fig. 5:**
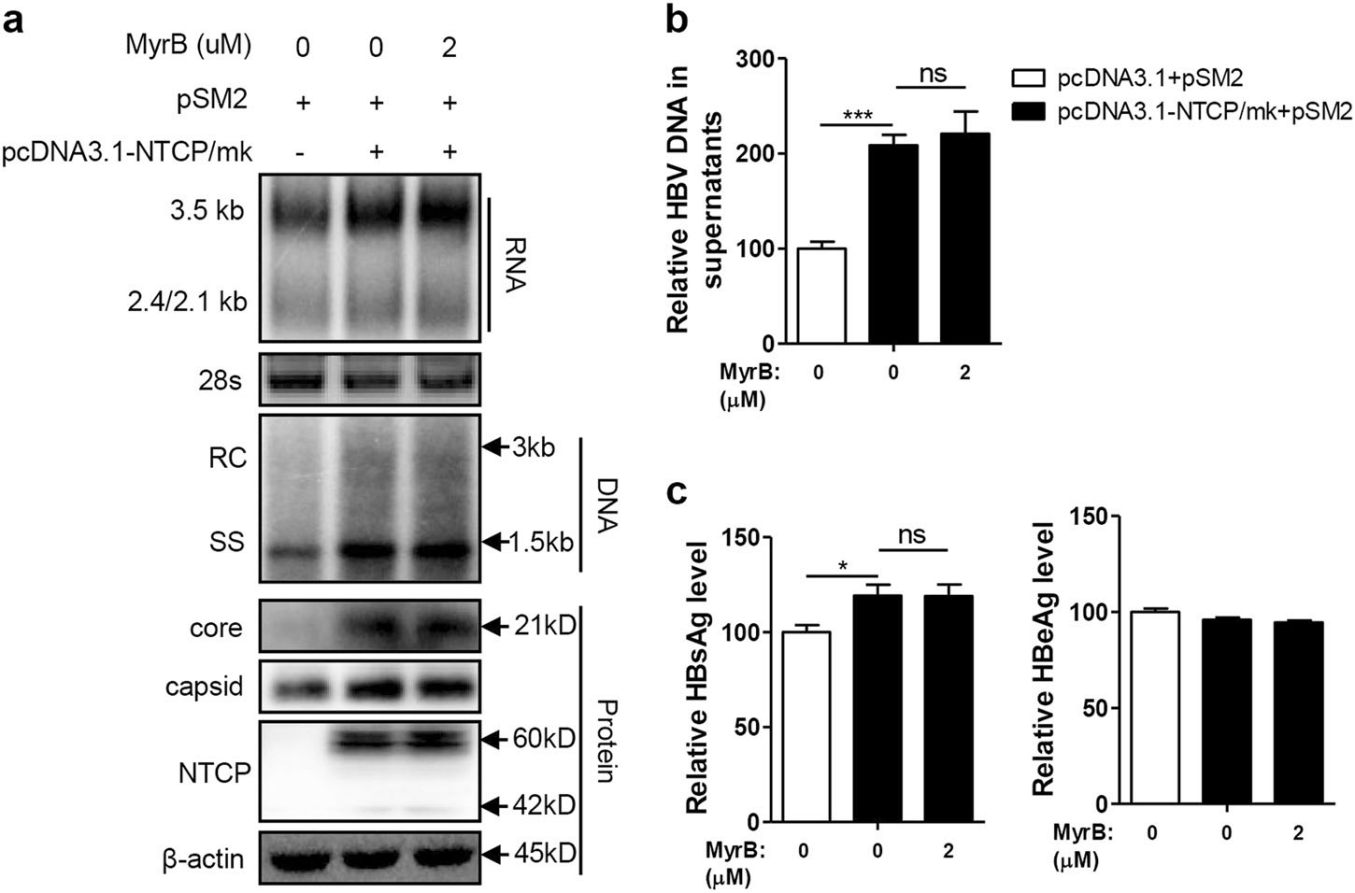
MyrB cannot block the activation of HBV replication by NTCP/mk. Huh7 cells transfected with the indicated plasmids were treated with 2 μM. **a** HBV transcription and replication intermediates were detected by northern blotting and Southern blotting, respectively (top panel). Expression of NTCP and core proteins and capsid formation in cells were analyzed by western blotting (bottom panel). **b** HBV DNA in culture supernatants were detected by real-time PCR (*n* = 4). **c** HBsAg and HBeAg in culture supernatants were detected by ELISA (*n* ≥ 4). A two-tailed *t*-test was used to determine differences in multiple comparisons. *P < 0.05, ***P < 0.001, ns: *P* > 0.05

In addition to blocking HBV entry, MyrB can bind to NTCP to inhibit BA uptake mediated by NTCP, as shown by previous reports^15,17^ and our data (Fig. S5a and b). Thus, we suspect that MyrB may counteract the NTCP-mediated upregulation of HBV replication by inhibiting NTCP-mediated BA uptake. We employed the mutated variant NTCP/mk to test this hypothesis. NTCP/mk retained the function of BA uptake (Fig. S5c), which was not blocked by MyrB due to the inability of the NTCP/mk to bind to the MyrB (Fig. S5a)^15^. In this setting, the upregulation of HBV replication by NTCP/mk was not affected by MyrB treatment (Fig. 5). These results demonstrated that MyrB abolished the NTCP-mediated upregulation of HBV replication by inhibiting the BA uptake.

### NTCP-mediated BA uptake specifically enhanced the activity of the HBV EN2/core promoter through FxRα

**Fig. 6:**
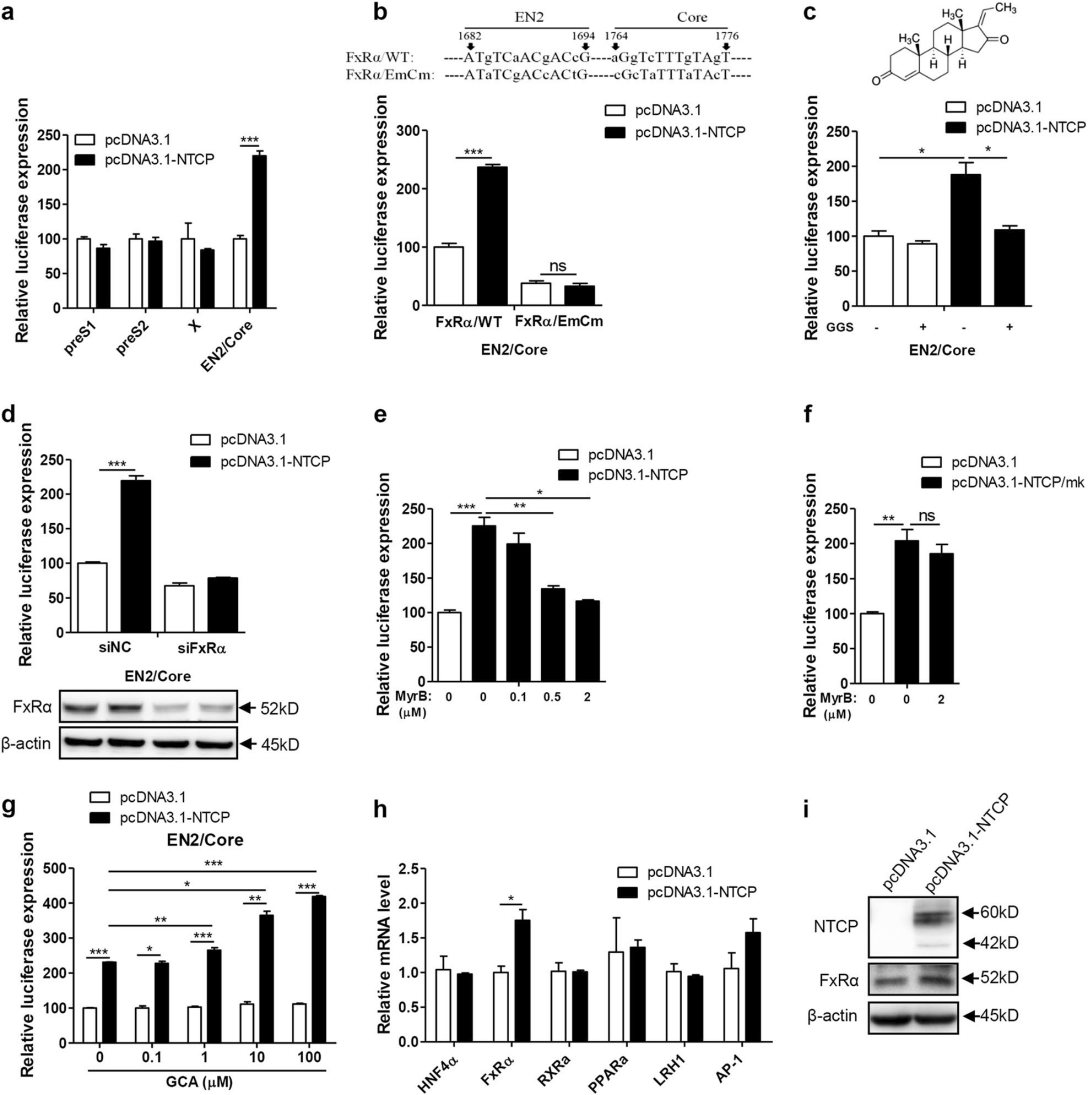
NTCP specifically improves activity of the HBV EN2/core promoter through FxRα. Huh7 cells transfected with the indicated plasmids were harvested. **a** Firefly luciferase activity was determined at 48 hpt. **b** Partial sequence of the construct pGL3-EN2/core (FxRα/WT) and the mutant construct of pGL3-EN2/core-FxRα/EmCm showed the FxRα binding sites and mutations introduced in EN2 (nts 1682 to 1694) and core (nts 1764 to 1776) promoter regions (top panel). Huh7 cells transfected with the indicated plasmids were harvested to determine firefly luciferase activity at 48 hpt (bottom panel). **c** Chemical structure of the FxRα antagonist GGS (top panel). Huh7 cells transfected with the indicated plasmids were treated with or without 10 µM GGS and then harvested to determine firefly luciferase activity at 48 hpt (bottom panel). **d** Huh7 cells transfected with the indicated plasmids and siRNAs were harvested to determine firefly luciferase activity at 48 hpt (top panel). The expression of FxRα was detected by western blotting (bottom panel). **e–g** Huh7 cells transfected with the indicated plasmids were treated with MyrB or GCA and then harvested to determine firefly luciferase activity at 48 hpt. **h** Total RNAs were extracted, and the mRNA levels of several transcription factors were detected by qRT-PCR. mRNA levels were normalized to *β-actin* mRNA. **i** The expression of FxRα was analyzed by western blotting. A two-tailed t-test was used to determine differences in multiple comparisons (*n* = 3). *P < 0.05, **P < 0.01, ***P < 0.001, ns: *P* > 0.05

According to the above results, we can infer that the observed upregulation of HBV by NTCP mainly depended on its BA-transporter function. Considering that FxRα is one of the most important sensing receptors for BAs^21–23^ and participates in HBV transcription via the regulation of HBV core promoter activity^13^, we hypothesized that FxRα may be involved in the mechanism by which the NTCP upregulates HBV replication. Therefore, we first analyzed the impact of NTCP expression on a series of HBV promoters and found only the activity of the HBV EN2/core promoter to be significantly augmented (Fig. 6a). To confirm the role of FxRα in this process, the two binding sites of FxRα in the EN2/core promoter were mutated^13^. As a result, the activity of the mutant EN2/core promoter was no longer enhanced by the NTCP expression (Fig. 6b), with the essential role of FxRα being inferred. Furthermore, we utilized guggulsterone Z (GGS), an FxRα antagonist, which had no toxic effect on cells (Fig. S4b). The GGS treatment completely offset the impact of the NTCP expression on the EN2/core promoter, as shown in Fig. 6c. In addition, when the expression of the FxRα was knocked down, the effect of the NTCP on the EN2/core promoter was obviously impaired (Fig. 6d). These results indicate that NTCP specifically enhanced HBV EN2/core promoter activity through FxRα.

To identify the role of NTCP-mediated BA uptake in this process, MyrB was applied as an uptake inhibitor, and the observed upregulation of EN2/core promoter activity by NTCP expression was counteracted by the MyrB treatment in a dose-dependent manner (Fig. 6e). Due to the NTCP/mk’s inability to bind to the MyrB, the upregulation of EN2/core promoter activity by the NTCP/mk was not affected by the MyrB treatment (Fig. 6f). Additionally, we found that glycocholic acid (GCA) enhanced the augmentation effect of the NTCP on EN2/core promoter activity in a dose-dependent manner (Fig. 6g), indicating that the NTCP specifically enhanced the activity of the HBV EN2/core promoter through FxRα, which was activated by NTCP-transported BAs.

Even BAs could directly active FxRα and enhance HBV core promoter activity. NTCP expression also specifically increased the FxRα mRNA (0.75-fold) (Fig. 6h) and protein levels (0.7-fold) (Fig. 6i) among known transcription factors for the HBV core promoter^13,24,25^.

### NTCP upregulated HBV transcription specifically through FxRα-mediated activation of the HBV EN2/core promoter

**Fig. 7:**
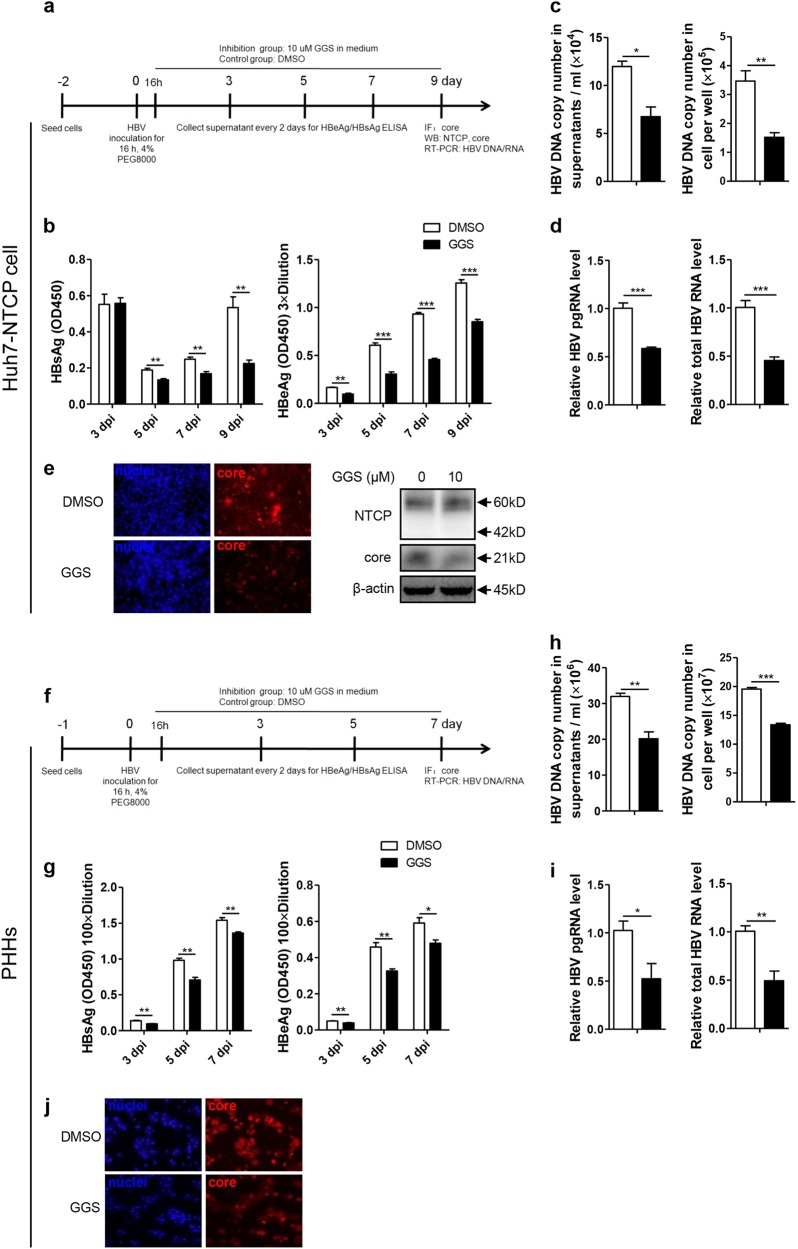
GGS inhibits HBV replication in Huh7-NTCP cells and PHHs. **a**, **f** Experimental layout: Huh7-NTCP cells and PHHs were severally seeded in 12-well plates and 48-wells plates and inoculated with HBV in the presence of 4% PEG8000 for 16 h. Next, the cells were cultured with culture medium added with 10 μM GGS or equivoluminal DMSO. **b**, **g** HBsAg and HBeAg were analyzed by ELISA (*n* ≥ 3). **c**, **h** Supernatant HBV DNA, intracellular HBV DNA, **d**, **i** HBV pgRNA and total RNA were detected by real-time PCR (*n* ≥ 3). **e**, **j** Cells were fixed and immunostained with an antibody against the core protein (red) (magnification ×200); **e** intracellular core protein was also detected by western blotting. A two-tailed *t*-test was used to determine differences in multiple comparisons. *P < 0.05, **P < 0.01, ***P < 0.001

**Fig. 8:**
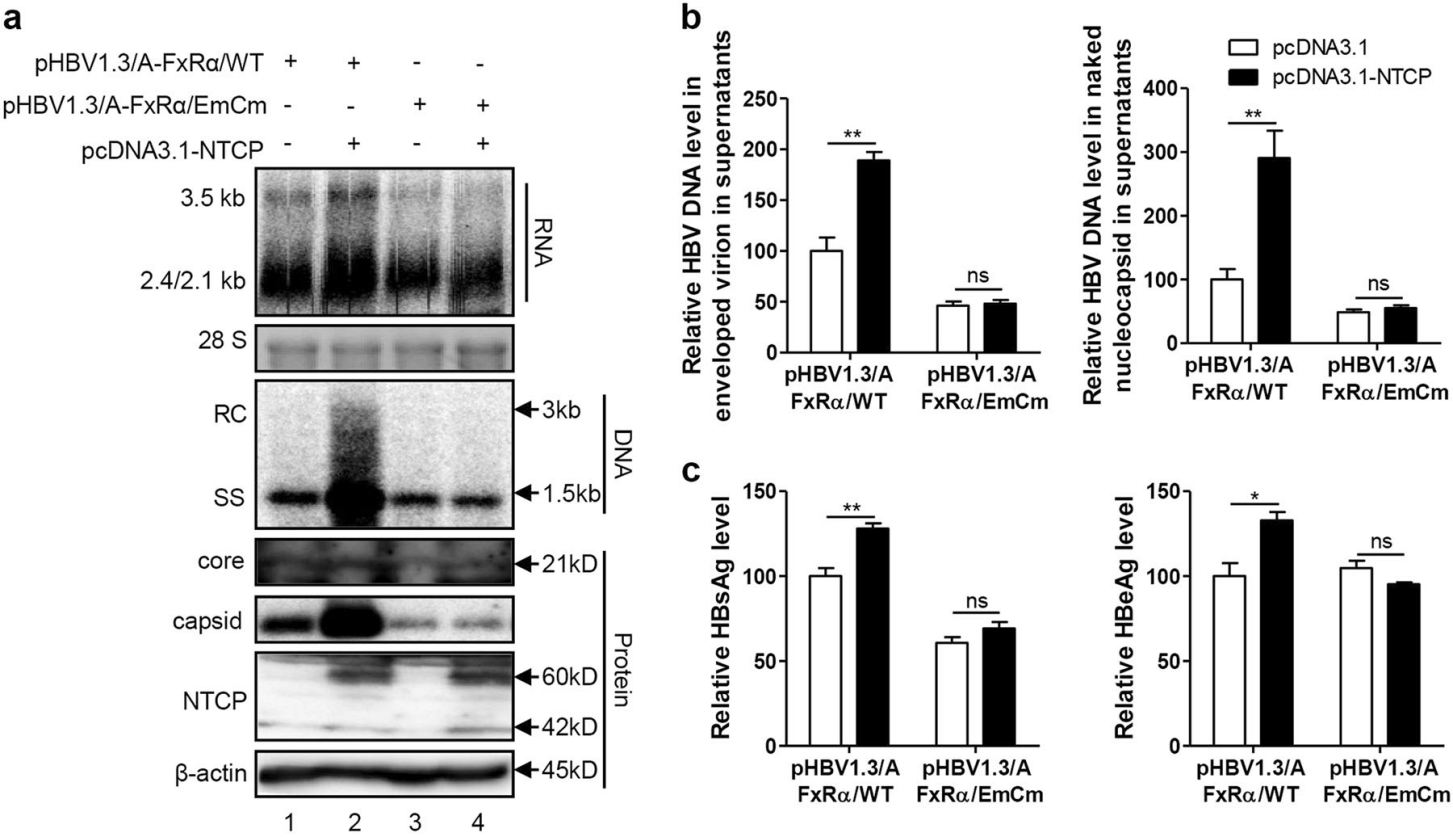
NTCP is unable to upregulate HBV replication with FxRα binding sites mutations. Huh7 cells transfected with the indicated plasmids were harvested at 72 hpt. **a** HBV transcription and replication intermediates were detected by northern blotting and Southern blotting, respectively (top panel). Expression of NTCP and core proteins and capsid formation in cells were analyzed by western blotting (bottom panel). **b** HBV DNA in enveloped virions and naked nucleocapsids in culture supernatants were detected by real-time PCR (*n* ≥ 3). **c** HBsAg and HBeAg in culture supernatants were detected by ELISA (*n* = 3). A two-tailed *t*-test was used to determine differences in multiple comparisons. *P < 0.05, **P < 0.01, ns: *P* > 0.05

We further employed GGS to assess whether NTCP specifically upregulates HBV transcription through FxRα in the Huh7-NTCP cells and in the primary human hepatocytes (PHHs) infected with HBV (Fig. 7a, f). GGS significantly reduced the levels of secreted HBsAg and HBeAg in both cells (Fig. 7b, g). Consistently, levels of both extracellular and intracellular HBV DNA, pgRNA and total RNA were also markedly decreased (Fig. 7c, d, h, i). Immunofluorescence (Fig. 7e, j) and western blotting (Fig. 7e) assays showed that the expression levels of the core proteins were significantly decreased after GGS treatment. Consistent results were also obtained in experiments involving Huh7 cells transfected with the HBV expression plasmid in (Fig. S6a-c) and HepG2.2.15 cells with stable HBV expression (Fig. S6d-f). To validate the specific role of FxRα in NTCP-dependent upregulation of HBV transcription, FxRα binding site mutations were introduced into the HBV genome (Fig. 8). As expected, the NTCP expression cannot increase the HBV RNA, DNA and protein levels any more (Fig. 8). Therefore, the NTCP specifically upregulated the HBV transcription by activating the HBV EN2/core promoter through the action of the FxRα.

## Discussion

NTCP has been shown to act as a functional receptor and major host-specific restriction factor for HBV infection^4,26^. As a peptidic NTCP inhibitor, MyrB is widely assumed to be an HBV entry inhibitor^6,8^, and its efficacy in targeting HBV infection has been evaluated clinically^9,10^. In the present study, NTCP was found to upregulate the HBV replication independent of its receptor function but does so depending on its BA-transporter function. We found that NTCP preferentially transported glycine- and taurine-conjugated BAs such as GCA-activated FxRα to upregulate HBV transcription, and MyrB abolished this NTCP-mediated upregulation of HBV replication both in vitro and in vivo. To our knowledge, this study is the first to demonstrate the role of NTCP in HBV replication at the postentry step and the potential antiviral activity of MyrB as an HBV replication inhibitor.

The physiological function of NTCP, as a key BA transporter, is to transport BAs from the portal blood into hepatocytes, a process that is important for maintaining BA homeostasis^11^. The amino acids of NTCP that have been reported as critical for efficient viral infection also contribute to BA binding^15^. Nonetheless, different from the sodium concentration dependency for transporting substrates, NTCP binding to HBV pre-S1 is largely independent of the sodium gradient between the extracellular and intracellular compartments^15^. Furthermore, both monkey and mouse NTCP are capable of transporting BAs, even though they do not support HBV and HDV infection^19,27^. According to our results, mutated NTCP/mk, with the capacity to transport BAs, did not function as an HBV receptor (Fig. S5)^15^. Thus, NTCP’s physiological function of transporting BAs may not be required for its role in mediating viral entry. Here, we show that NTCP upregulated HBV replication at the postentry step and that this depended on the continued functioning of NTCP as the means of transport for the BAs. Therefore, NTCP is involved in HBV entry as well as transcription.

BAs act as natural ligands of FxRα and thus directly activate FxRα^21,22^; furthermore, they enhance HBV core promoter activity^13^. Thus, NTCP-transported BAs could directly activate the FxRα, and they upregulated the HBV transcription. In addition to FxRα, BAs can activate a range of other nuclear receptors (NRs) and thus regulate the expression of genes involved in many biological processes^12,23^. Overexpression of NTCP increased the transcription FxRα but only slightly. Thus, the significant effect of NTCP on HBV transcription mainly resulted from the direct activation of FxRα by NTCP-transported BAs. SIRT1 regulated the FxRα activity^28^_,_ and the effect of FxRα on the HBV transcription was substantially enhanced by SIRT1^29,30^. Whether NTCP might affect the FxRα activation by SIRT1 is under investigation.

Although previous reports have shown that chenodeoxycholic acid (CDCA) activates the HBV EN2/core promoter through FxRα in the Huh7 cells^13^, the effect appears to be independent of NTCP expression because the expression level of NTCP is very low in the Huh7 cells^4^ and CDCA uptake occurs primarily through simple diffusion^31^. We also found that CDCA increased the activity of the HBV EN2/core promoter in a dose-dependent manner regardless of whether the NTCP was expressed (Fig. S7a). Compared with unconjugated BAs such as CDCA, NTCP prefers to transport glycine- and taurine-conjugated BAs such as GCA^32^. In agreement with this report, we found that GCA enhanced EN2/core promoter activity, which was dependent on the NTCP expression (Fig. 6g). Hence, our data showed that GCA was transported by NTCP-activated FxRα and thus upregulated HBV transcription, which is quite different from the previous reports that had suggested unconjugated BAs such as CDCA activated FxRα and thus upregulated HBV replication^21^. Overall, it would be interesting to identify whether other conjugated BAs preferentially transported by NTCP also play a similar role during this process.

Because of NTCP’s key role in mediating HBV entry, the peptidic NTCP inhibitor MyrB has been recognized as an effective HBV entry inhibitor^6,8^, and its efficacy in targeting HBV infection has been evaluated clinically^9,10^. Notably, MyrB can also inhibit the BA transport mediated by NTCP^15^. Thus, although mouse NTCP does not support HBV entry^19^, MyrB still suppresses HBV replication in an HBV hydrodynamic injection mouse model (Fig. 4). We also found that although HBV replication was not affected in the first 3 days post infection, impairment began on day 5 post infection (Fig. 3b), indicating that only sustained MyrB treatment efficiently inhibited HBV replication. Accordingly, the effect of MyrB on viral replication cannot be detected when the cells are incubated with MyrB for only 16 h^33^. Interestingly, recent studies have revealed that MyrB treatment significantly affects plasma BA levels in healthy volunteers^9,34^, further hinting at the possibility of MyrB as an HBV replication inhibitor because the inhibition of BA uptake may help to suppress HBV replication, as shown in our study. In addition, because MyrB inhibits HBV replication by inhibiting NTCP-mediated BA transport, the antiviral efficacy of MyrB was dependent on the expression of NTCP (Fig. 2). Considering that NTCP expression dropped sharply in ex vivo-cultured PHHs (Fig. S8f)^17,27,35^, the inhibitory effect of MyrB on HBV replication in PHHs was not observed (Fig. S8).

In conclusion, our study demonstrated that in addition to the entry step, NTCP is able to upregulate HBV replication independent of its receptor function, which can be offset by the specific NTCP-targeting entry inhibitor MyrB. These results suggest that the drug MyrB could potentially target HBV, and HDV entry may also possess antiviral activity toward intracellular HBV. These findings further broaden our knowledge regarding the role of NTCP in the HBV life cycle. Additionally, our results provide a reference for developing anti-HBV agents targeting NTCP and for comprehensively assessing the therapeutic effect of such specific NTCP inhibitors as MyrB.

## Materials and methods

### Cell culture and HBV infection

Huh7, Huh7-NTCP, and HepG2.2.15 cells were cultured as described previously^36^. The HepG2.2.15-NTCP cell line stably expressing human NTCP (hNTCP) was generated by infecting HepG2.2.15 with recombinant lentivirus, as described previously^37^. Primary human hepatocytes (PHHs) were provided by XenoTech (Kansas City, KS, USA) and cultured according to the manufacturer’s instructions. These cells were all confirmed to be mycoplasma-negative.

For HBV infection, 5 × 10^5^ Huh7-NTCP cells were seeded in 12-well plates, or 1.5 × 10^5^ PHHs were seeded in 48-well plates for 4 h. Next, the medium was changed with fresh culture medium with 2% DMSO and left for another 24 h. The cells were infected by inoculation with ~500 genome equivalents (ge) per cell of HBV in the presence of 4% PEG8000. After 16 h, the infection inoculum was removed, and the cells were washed three times with phosphate-buffered saline (PBS) and maintained subsequently in culture medium with 2% DMSO and 2 μM MyrB or 10 μM GGS. The medium was changed every 2 days.

### HBV capsid extraction and native agarose gel electrophoresis

The HBV capsids in the cells were extracted and detected as described previously^36^. Briefly, the cells were harvested with 200 μl-specific lysis buffer (10 mM Tris, pH 7.5, 1 mM EDTA, 50 mM NaCl, 8% sucrose, and 0.25% NP-40) and incubated on ice for 10 min. The samples were centrifuged for 10 min at 13,000 rpm and 4 °C. The supernatants were incubated with 6μM MgCl_2_, 40μg DNaseI and 300μg RNase for 20 min at 37 °C, followed by centrifugation for 10 min at 13,000 rpm and 4 °C. The supernatants were resolved by 1.6% native agarose gel electrophoresis for 2.5 h (volt = 45 V) at 4 °C. A nitrocellulose membrane was used for capsid transfer via adsorption overnight. The anti-Core (DAKO) antibody was used for capsid detection.

### Southern blotting and northern blotting

Total cellular RNA was extracted using TRIzol reagent (Ambion, Carlsbad CA, USA) according to the manufacturer’s instructions and subjected to northern blotting as described previously^36^. Encapsidated HBV replication intermediates in the cells were extracted and subjected to Southern blotting as described previously^36^.

### Real-time PCR

One-step quantitative reverse transcription-polymerase chain reaction (qRT-PCR) was performed to analyze specific mRNAs. HBV DNA in cell culture supernatants was extracted and detected using real-time PCR. The primers used are listed in Supplementary Table 1.

### Hydrodynamic injection in mice

Male C57BL/6 (H-2b) mice (5–6 weeks of age) were used, and the hydrodynamic-based HBV mouse model was employed as described previously^20,38^. This study was performed in strict accordance with the recommendations in the Guide for the Care and Use of Laboratory Animals according to the regulations in the People’s Republic of China. All animal experiments were approved by the Institutional Animal Ethical Committee of WIV, CAS (Serial number: WIVA02201701). Mice were kept under specific-pathogen-free (SPF) conditions in the Central Animal Laboratory of the Wuhan Institute of Virology, Chinese Academy of Sciences (WIV, CAS. License number: SYXK2014-0034). All animals in these studies were maintained under the following conditions: 5–6 animals per cage (Sealsafe^TM^, Tecniplast, Varese, Italy), controlled temperature: 22 ± 1 °C, controlled relative humidity: 55 ± 15%, air conditioning: air filtered through a HEPA filter, 12 h-light/dark cycle (0800–2000 h light), diet: SPF rat maintenance feed (Keaoxieli, Beijing, China). The mice were anesthetized with isoflurane and then euthanized by cervical dislocation at the experimental endpoint.

Male mice (8 mice each group), randomly divided into the control group and treatment groups, were hydrodynamically injected with 14 μg pSM2 via the tail vein within 5–7s^20^. MyrB and control peptide were reconstituted in water and diluted with PBS to reach a final concentration of 50 μg/ml. The 5 μg dose was administered twice daily (0900 and 2100) by subcutaneous injection, with each injection containing 2.5 μg peptide. Mice were euthanized by cervical dislocation at 4 days posthydrodynamical injection (dphi). The levels of HBeAg, HBsAg and HBV DNA in the serum were detected by ELISA daily. For analysis of the serum HBV DNA, serum samples were first digested overnight with DNase I at 37 °C to remove the HBV plasmid pSM2, followed by extraction. The HBV DNA extracted from 100 mg liver sections at 4 dphi was subjected to Southern blotting assays. The total RNAs extracted from the liver sections at 4 dphi were subjected to a qRT-PCR assay to assess the levels of HBV pgRNA and HBV total RNAs. The primers used are listed in Supplementary Table 1. The HBV RNAs were also detected by northern blotting assay. Intrahepatic core protein were analyzed by immunohistochemical staining as described previously^38,39^.

### Statistical analysis

Statistical analysis was carried out using GraphPad Prism 5.0 software (GraphPad Software). The results are presented as the mean ± standard deviations (*n* ≥ 3). The statistical significance of the differences in multiple comparisons was determined using Student’s *t*-test.

For further details regarding the methods used, please refer to the supplementary materials and methods.

## Electronic supplementary material

Supplementary Materials and Methods

Supplementary Figure 1a-c

Supplementary Figure 2a-c

Supplementary Figure 3a-c

Supplementary Figure 4a-b

Supplementary Figure 5a-c

Supplementary Figure 6a-f

Supplementary Figure 7a-b

Supplementary Figure 8a-f

Supplementary Table 1

Supplementary Table 2

Supplementary Table 3

## Data Availability

The authors declare that all relevant data are available from the corresponding author upon request.
