# Supplementary material for: Upregulation of HBV transcription by sodium taurocholate cotransporting polypeptide at the postentry step is inhibited by the entry inhibitor Myrcludex B
Source: Emerg Microbes Infect. 2018 Nov 21;7:186. doi: 10.1038/s41426-018-0189-8 (PMC6246608; doi:10.1038/s41426-018-0189-8)
Supplement: Supplementary file 1 — Supplementary Materials and Methods [file 41426_2018_189_MOESM1_ESM.doc]

**Supplementary Materials and Methods**

**Cell culture and transfection**

The human hepatocellular carcinoma cell line Huh7 (kindly provided by Prof. Mengji Lu (University Hospital of Essen, Essen, Germany) was cultured in Dulbecco’s modified Eagle’s medium (DMEM) (Life Technologies) supplemented with 10% fetal bovine serum (FBS, containing 8.9 μmol/L of bile acids) (Life Technologies) and 100 U penicillin/streptomycin (Life Technologies)/ml. The HepG2.2.15 cell line with an integrated dimer of the HBV genome (GenBank accession number: U95551) was kindly provided by Prof. Mengji Lu (University Hospital of Essen, Essen, Germany) and cultured in RPMI medium (HyClone) supplemented with 10% FBS and 500 μg/ml G418 (Sigma-Aldrich). The HepG2.2.15-NTCP stable cell line expressing human NTCP was generated from HepG2.2.15 and cultured in RPMI medium supplemented with 10% FBS, 500 μg/ml G418 and 5 μg/ml puromycin. A Huh7-NTCP stable cell line expressing human NTCP was generated from Huh7 cells and maintained in DMEM supplemented with 10% FBS and 5 μg/ml puromycin. All cells were cultured at 37°C in a 5% CO_2_ humidified incubator with regular passaging every 2 to 3 days.

For cell transfection, Huh7 and Huh7-NTCP cells were seeded at approximately 30% confluence and transfected with Lipofectamine 2000 (Invitrogen) according to the manufacturer’s instructions. For most experiments, transfection was performed in 6-well plates, and 1.5 µg of every type of plasmid was used per well. For expression of NTCP in a dose-dependent manner, four doses (0, 0.5, 1.0, 1.5 µg per well) were used, and the control plasmid pcDNA3.1 was added up to 1.5 µg per well. For transfection of siRNAs, 20 nM per well was used. Huh7 and Huh7-NTCP cells were harvested at 72 h post-transfection. HepG2.2.15 and HepG2.2.15-NTCP cells were harvested at 120 h post-treatment.

**Peptide, antibodies, siRNAs and other reagents**

MyrB derived from the preS1 domain of HBV containing the 2-48 residues with amino-terminal myristoylation modification, MyrB-FITC with additional carboxyl-terminal FITC modification and control peptide without amino-terminal myristoylation modification were synthesized by GL Biochem (Shanghai, China). GGS and GCA were purchased from Sigma. Stock solutions of MyrB, GGS and GCA were prepared in DMSO, diluted extemporaneously in complete DMEM or RPMI media, and added to the cell cultures at the indicated concentrations. siRNAs targeting human NTCP and the control siRNA (siNC) were synthesized by GenePharma, and their target sequences are listed in Supplementary Table 2. Antibodies used in this study are listed in Supplementary Table 3.

**Construction of plasmids**

The coding sequence of human NTCP (GenBank accession number NM_003049) was amplified by PCR using the primers listed in Supplementary Table 1. The amplified gene was inserted into the pcDNA3.1 plasmid to generate pcDNA3.1-NTCP. The pcDNA3.1-NTCP/mk mutant construct and the luciferase reporter vectors pSP1, pSP2, pXP, pEN2/CP and pEN2/CP-EmCm were generated as described previously[^1-3^](#_ENREF_1). pRL-TK was purchased from Promega. Two previously described HBV replication-competent plasmids, pSM2 and pHBV1.3/A, were used. The plasmid pHBV1.3/A-FxRα/EmCm with FxRα binding site mutation was constructed based on pHBV1.3/A.

**Supernatant HBV DNA extraction and detection**

Supernatant HBV DNA extraction and detection were performed as described previously[^5^](#_ENREF_5). Briefly, mature viral particles and naked nucleocapsids were immunoprecipitated with mixed anti-HBs antibodies (A11, BJ11and S1) and anti-Core antibody, respectively. HBV DNA was then extracted using a QIAamp DNA Blood Mini Kit (QIAGEN, Germany) according to the manufacturer’s instructions and detected by real-time PCR.

**Luciferase and immunofluorescence assays**

For the luciferase assay, Huh7 cells were seeded at approximately 30% confluence in 24-well plates and transfected with 375 ng pcDNA3.1 or pcDNA3.1-NTCP or pcDNA3.1-NTCP/mk combined with 100 ng indicated reporter plasmids and 10 ng pRL-TK. Luciferase activity was detected at 48 h after transfection or reagent processed as described previously[^6^](#_ENREF_6). The results were normalized to *Renilla* luciferase activity. For the immunofluorescence (IF) assay, cells were plated on glass cover slips and stained as described previously[^5^](#_ENREF_5). An anti-Core antibody (Dako) was used as the primary antibodies, and an Alexa Fluor 568-conjugated antibody (Life Technologies) was used for secondary detection.

**References**

1 Zhang, X. Y. *et al.* Modulation of Hepatitis B Virus Replication and Hepatocyte Differentiation by MicroRNA-1. *Hepatology* **53**, 1476-1485, doi:10.1002/hep.24195 (2011).

2 Ramiere, C. *et al.* Transactivation of the hepatitis B virus core promoter by the nuclear receptor FXRalpha. *Journal of virology* **82**, 10832-10840, doi:10.1128/JVI.00883-08 (2008).

3 Yan, H. *et al.* Sodium taurocholate cotransporting polypeptide is a functional receptor for human hepatitis B and D virus. *eLife* **1**, e00049, doi:10.7554/eLife.00049 (2012).

4 Qin, B. *et al.* The amino acid substitutions rtP177G and rtF249A in the reverse transcriptase domain of hepatitis B virus polymerase reduce the susceptibility to tenofovir. *Antivir Res* **97**, 93-100, doi:10.1016/j.antiviral.2012.12.007 (2013).

5 Zhao, K. *et al.* Ceruloplasmin inhibits the production of extracellular hepatitis B virions by targeting its middle surface protein. *The Journal of general virology* **98**, 1410-1421, doi:10.1099/jgv.0.000794 (2017).

6 Chen, H. H. *et al.* An Alternative Splicing Isoform of MITA Antagonizes MITA-Mediated Induction of Type I IFNs. *J Immunol* **192**, 1162-1170, doi:10.4049/jimmunol.1300798 (2014).
