# Supplementary figures and images for: Upregulation of HBV transcription by sodium taurocholate cotransporting polypeptide at the postentry step is inhibited by the entry inhibitor Myrcludex B
Source: Emerg Microbes Infect. 2018 Nov 21;7:186. doi: 10.1038/s41426-018-0189-8 (PMC6246608; doi:10.1038/s41426-018-0189-8)

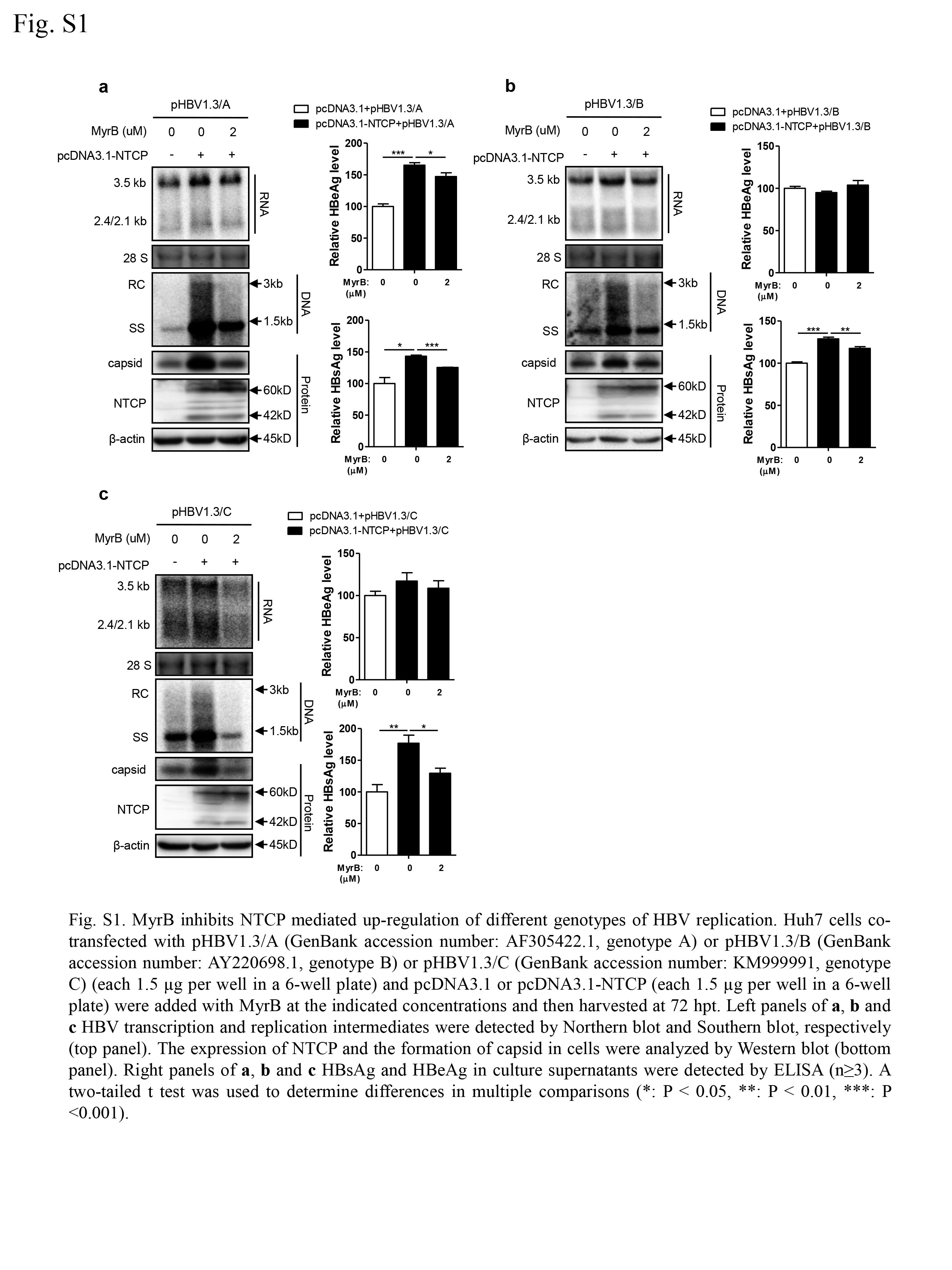

Supplement: Supplementary file 2 — Supplementary Figure 1a-c [file 41426_2018_189_MOESM2_ESM.jpg]

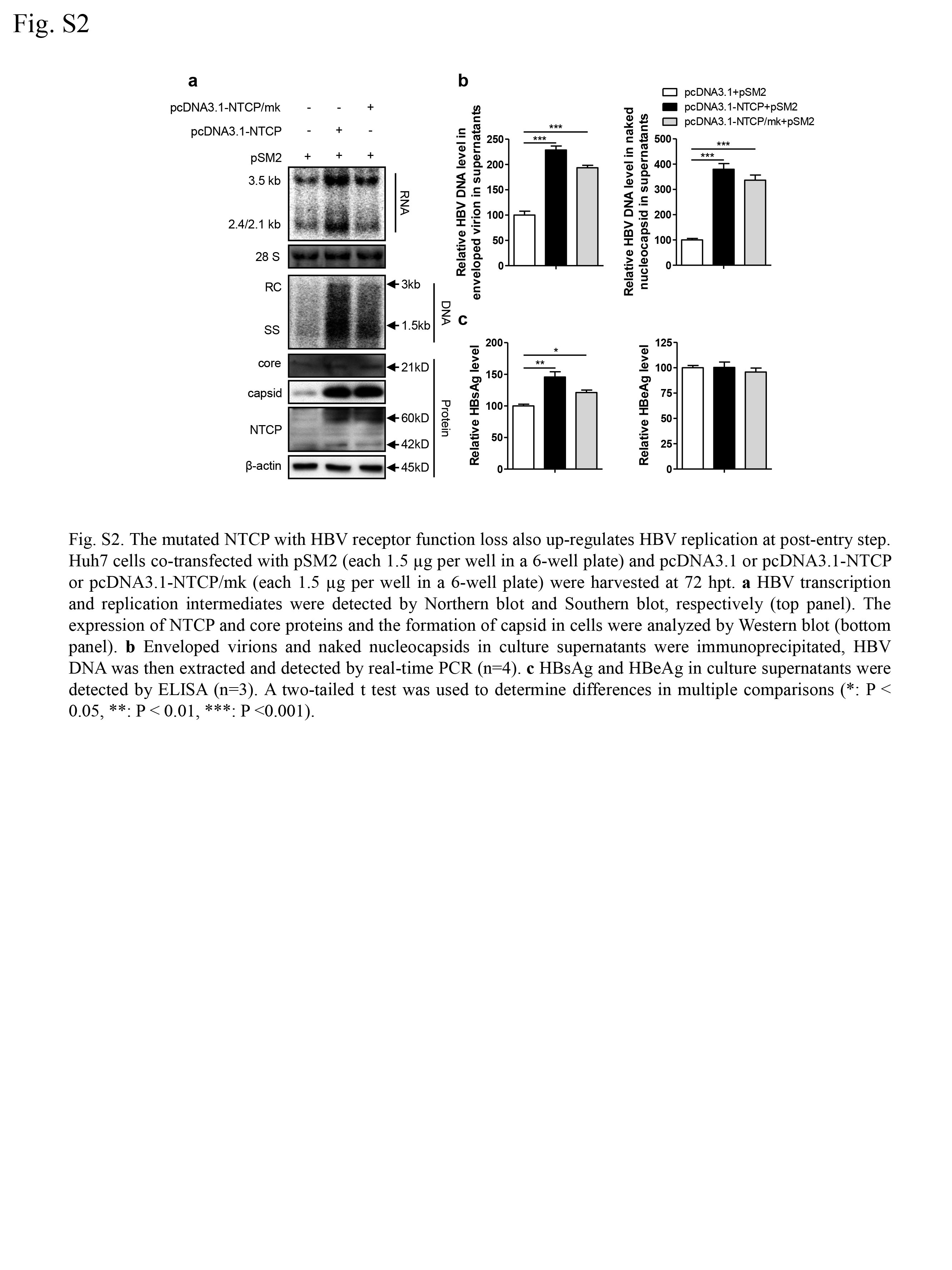

Supplement: Supplementary file 3 — Supplementary Figure 2a-c [file 41426_2018_189_MOESM3_ESM.jpg]

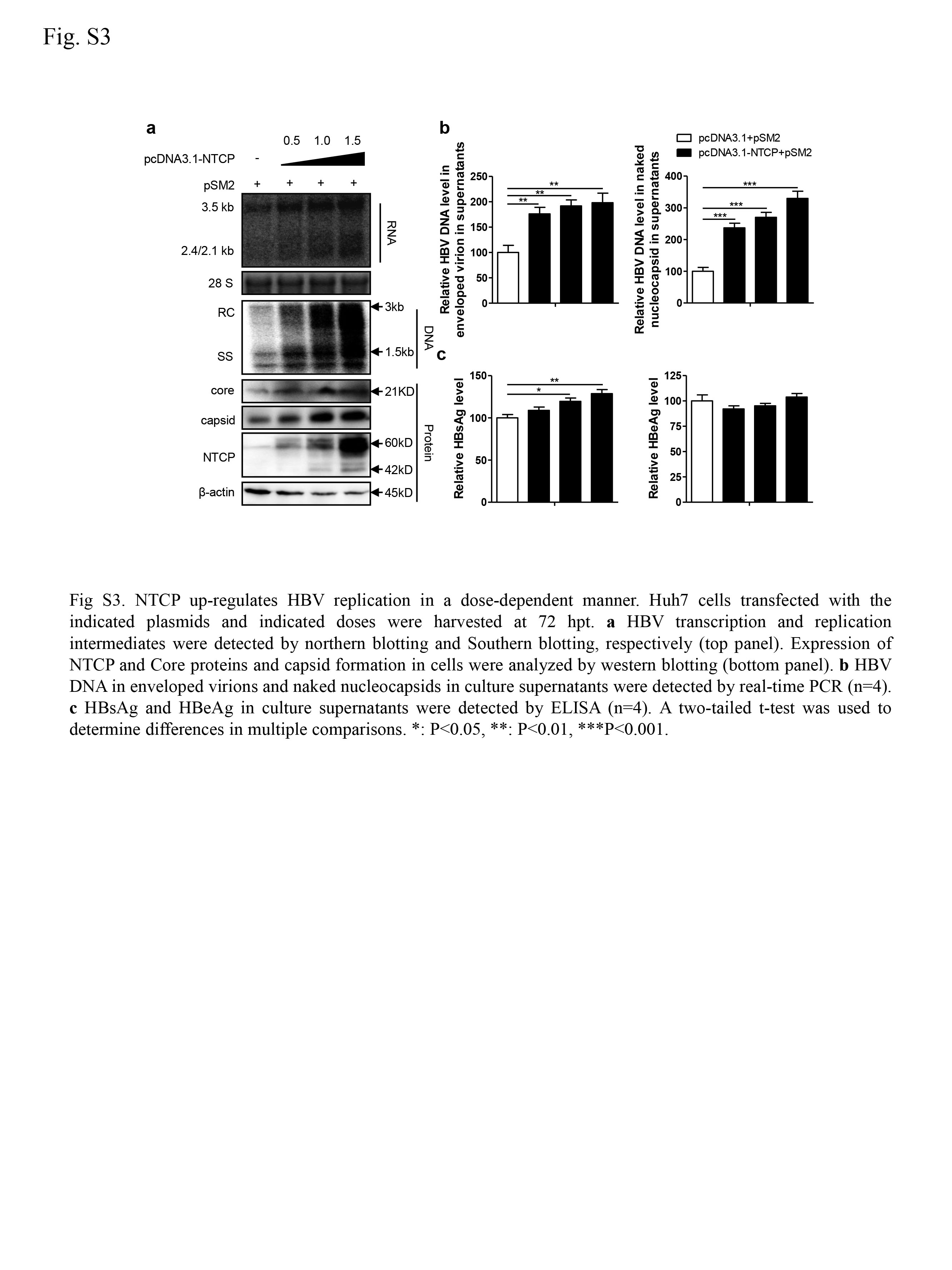

Supplement: Supplementary file 4 — Supplementary Figure 3a-c [file 41426_2018_189_MOESM4_ESM.jpg]

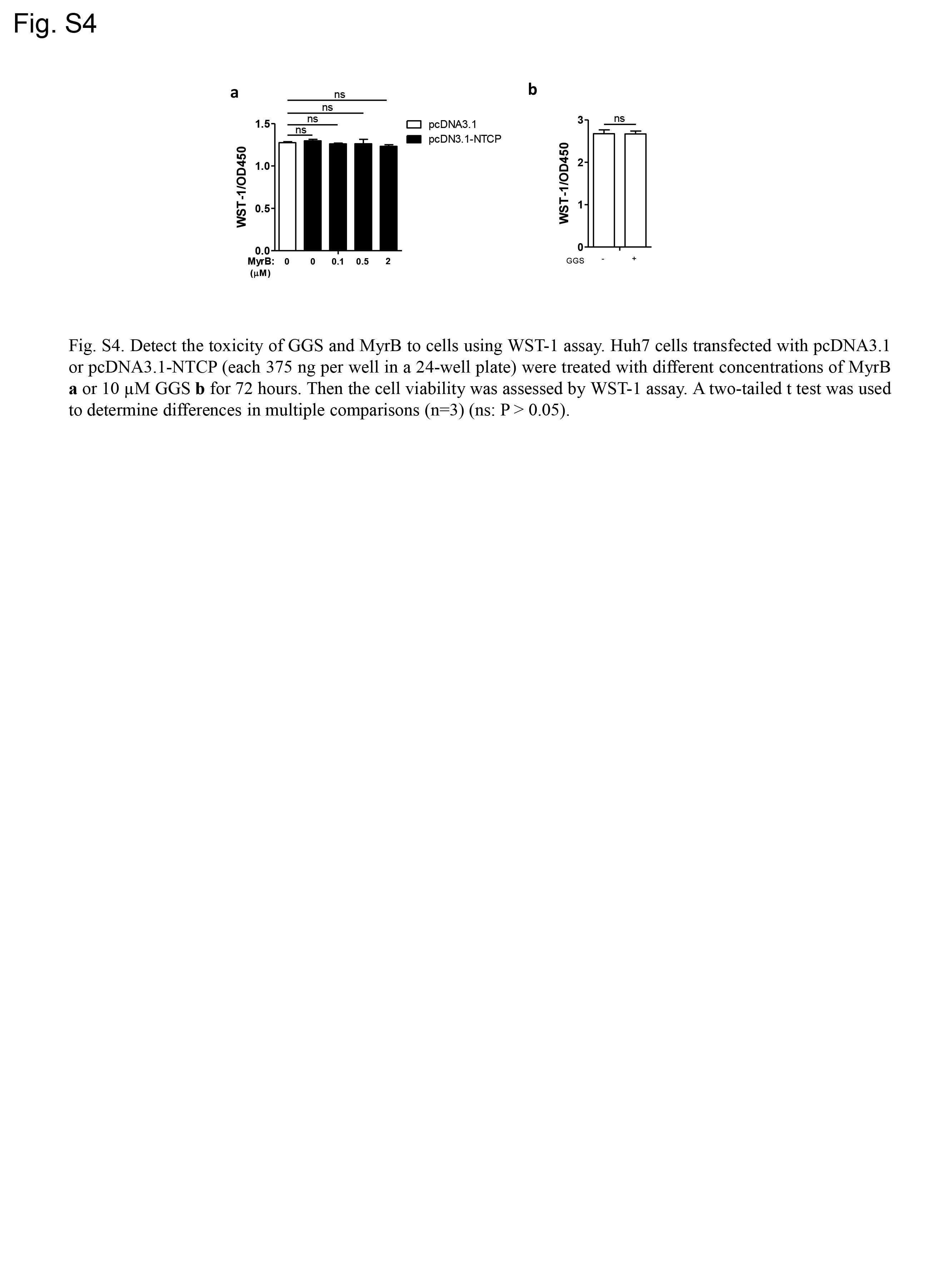

Supplement: Supplementary file 5 — Supplementary Figure 4a-b [file 41426_2018_189_MOESM5_ESM.jpg]

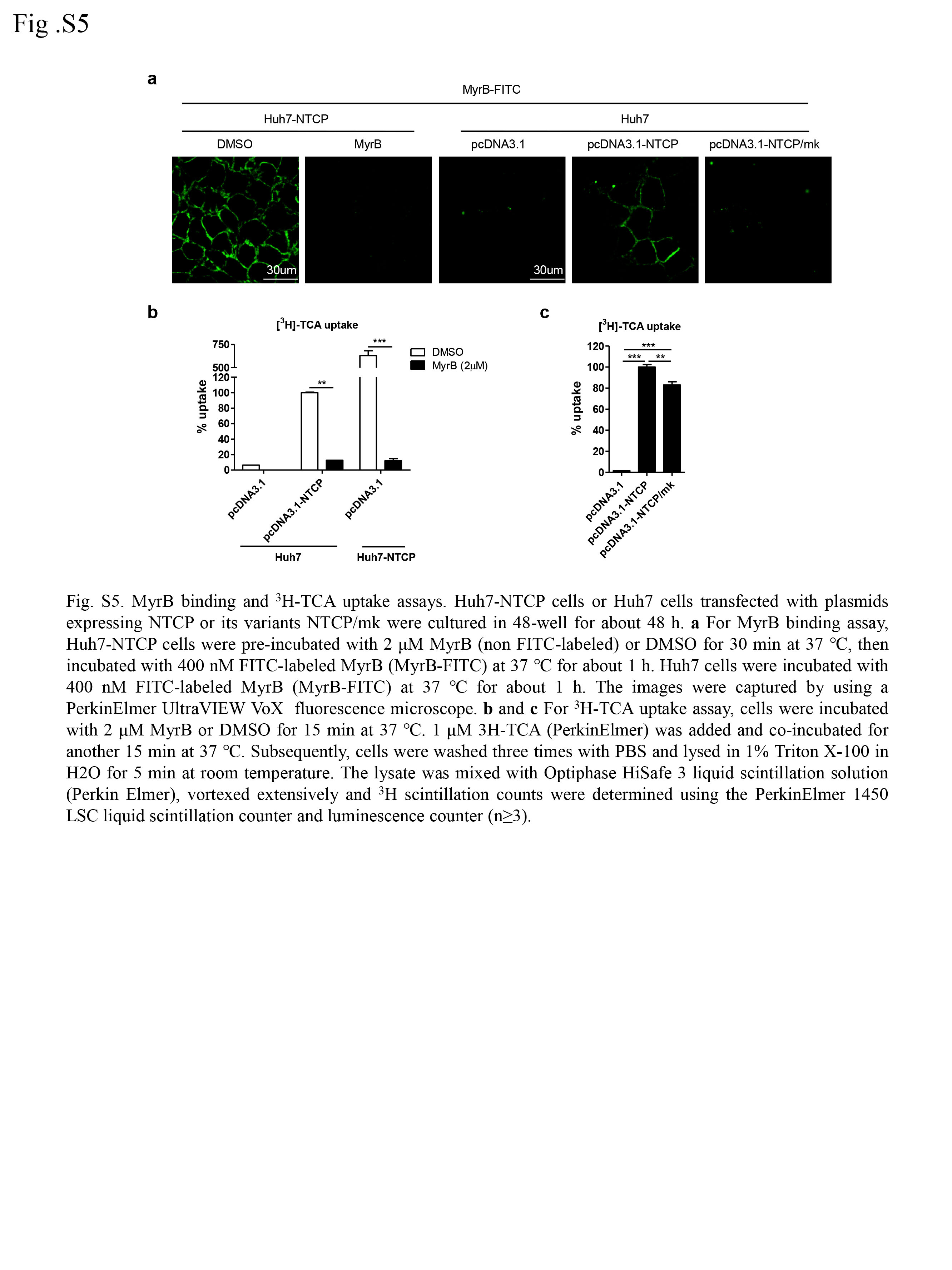

Supplement: Supplementary file 6 — Supplementary Figure 5a-c [file 41426_2018_189_MOESM6_ESM.jpg]

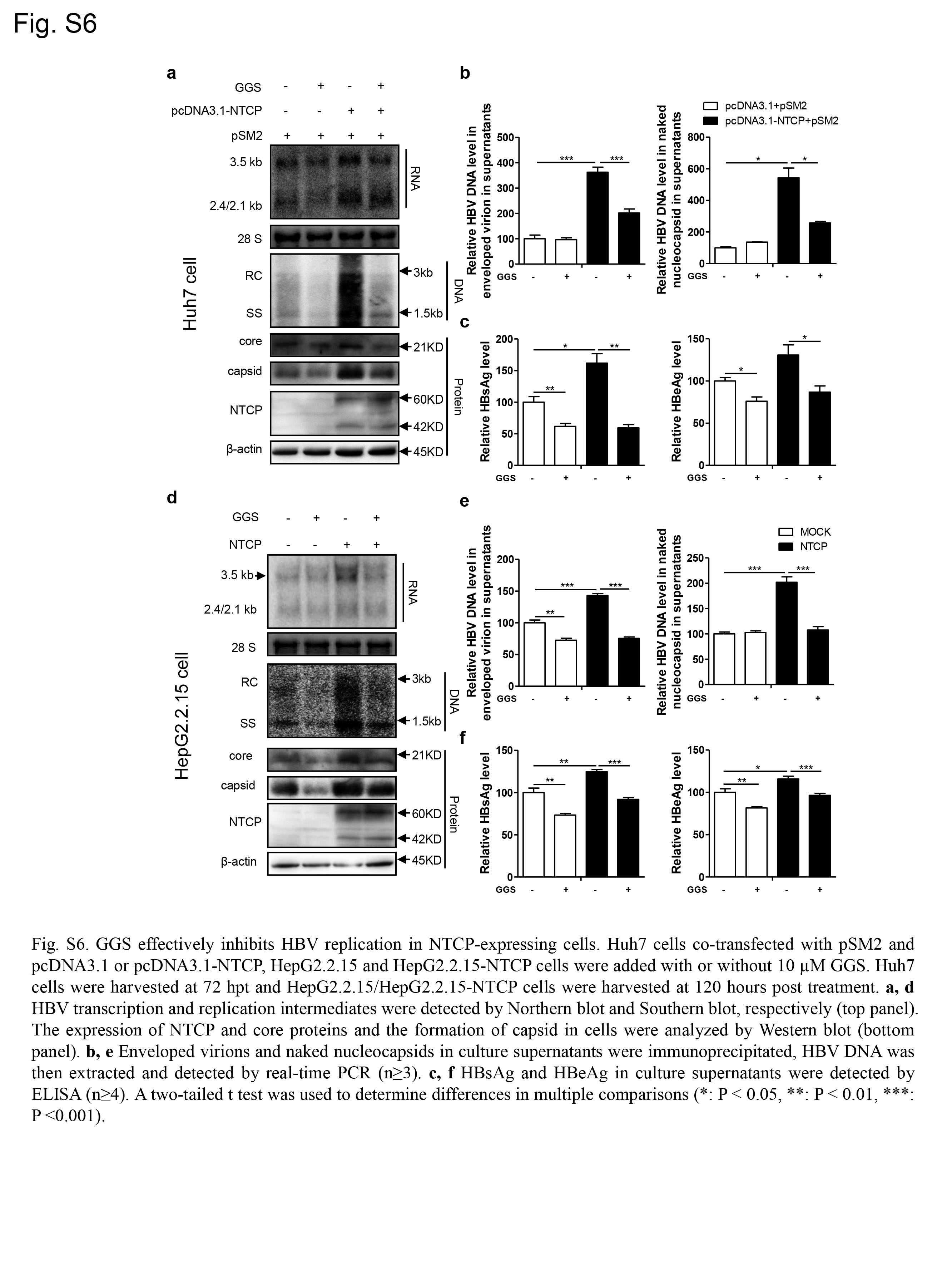

Supplement: Supplementary file 7 — Supplementary Figure 6a-f [file 41426_2018_189_MOESM7_ESM.jpg]

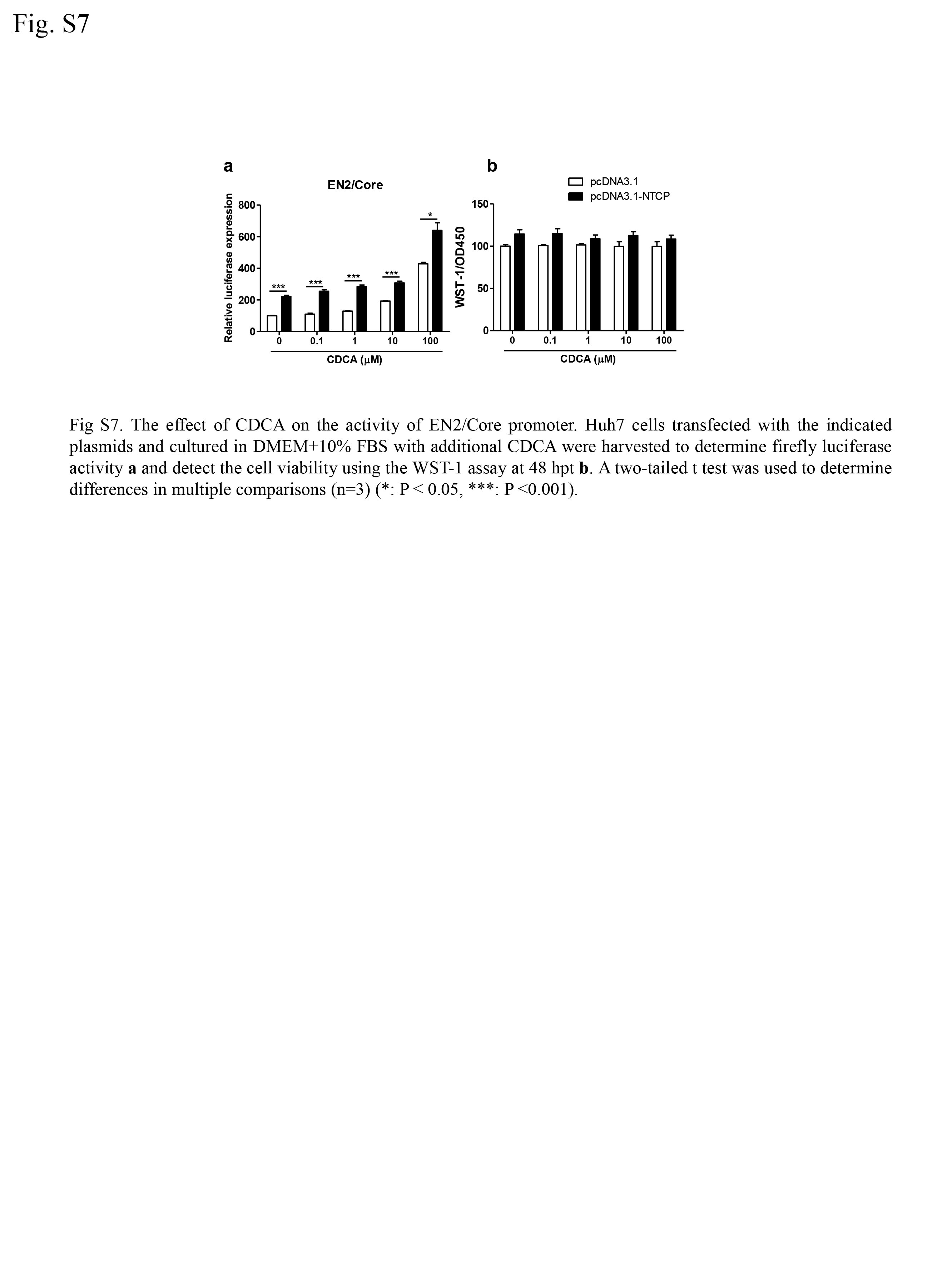

Supplement: Supplementary file 8 — Supplementary Figure 7a-b [file 41426_2018_189_MOESM8_ESM.jpg]

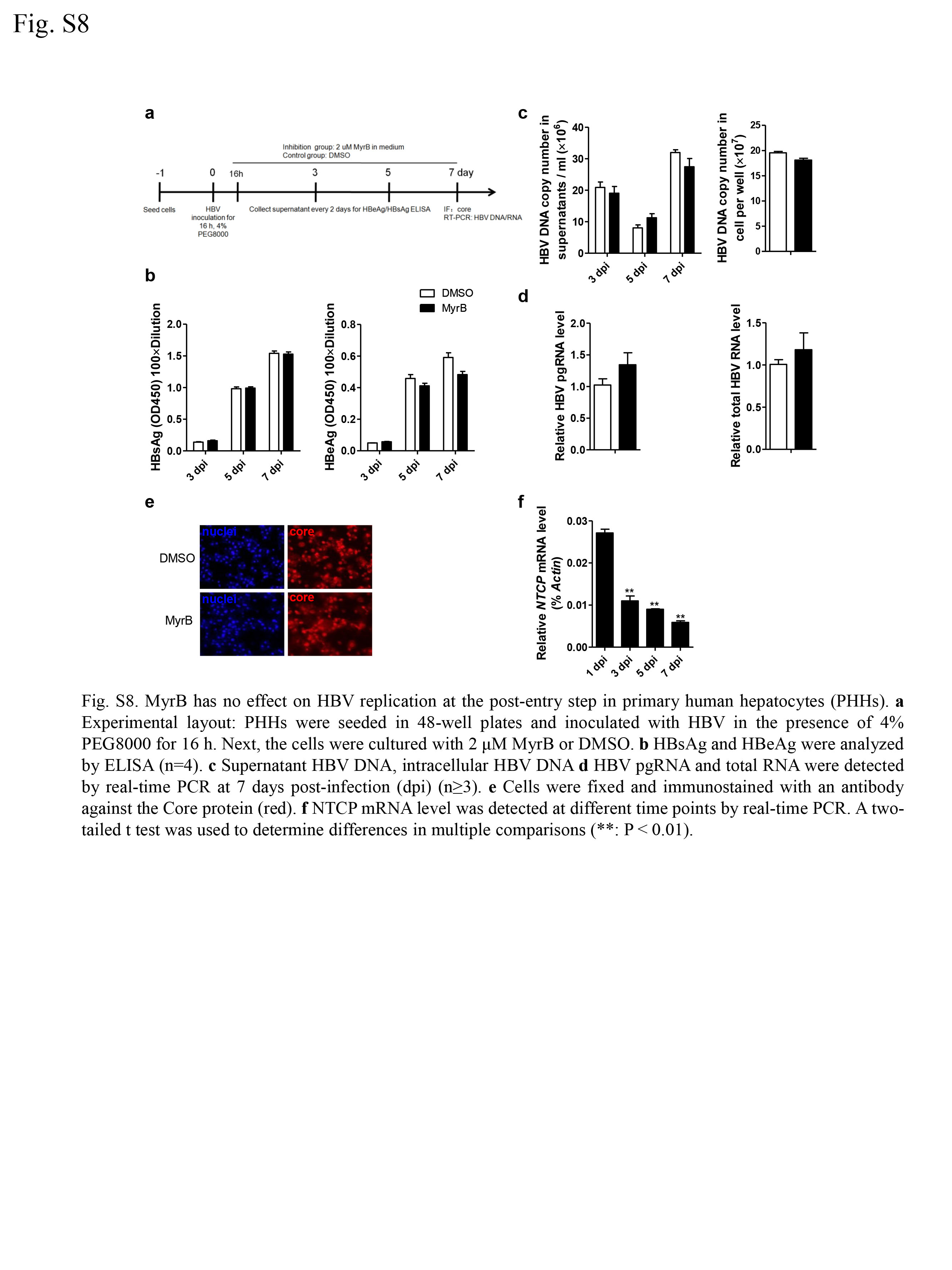

Supplement: Supplementary file 9 — Supplementary Figure 8a-f [file 41426_2018_189_MOESM9_ESM.jpg]

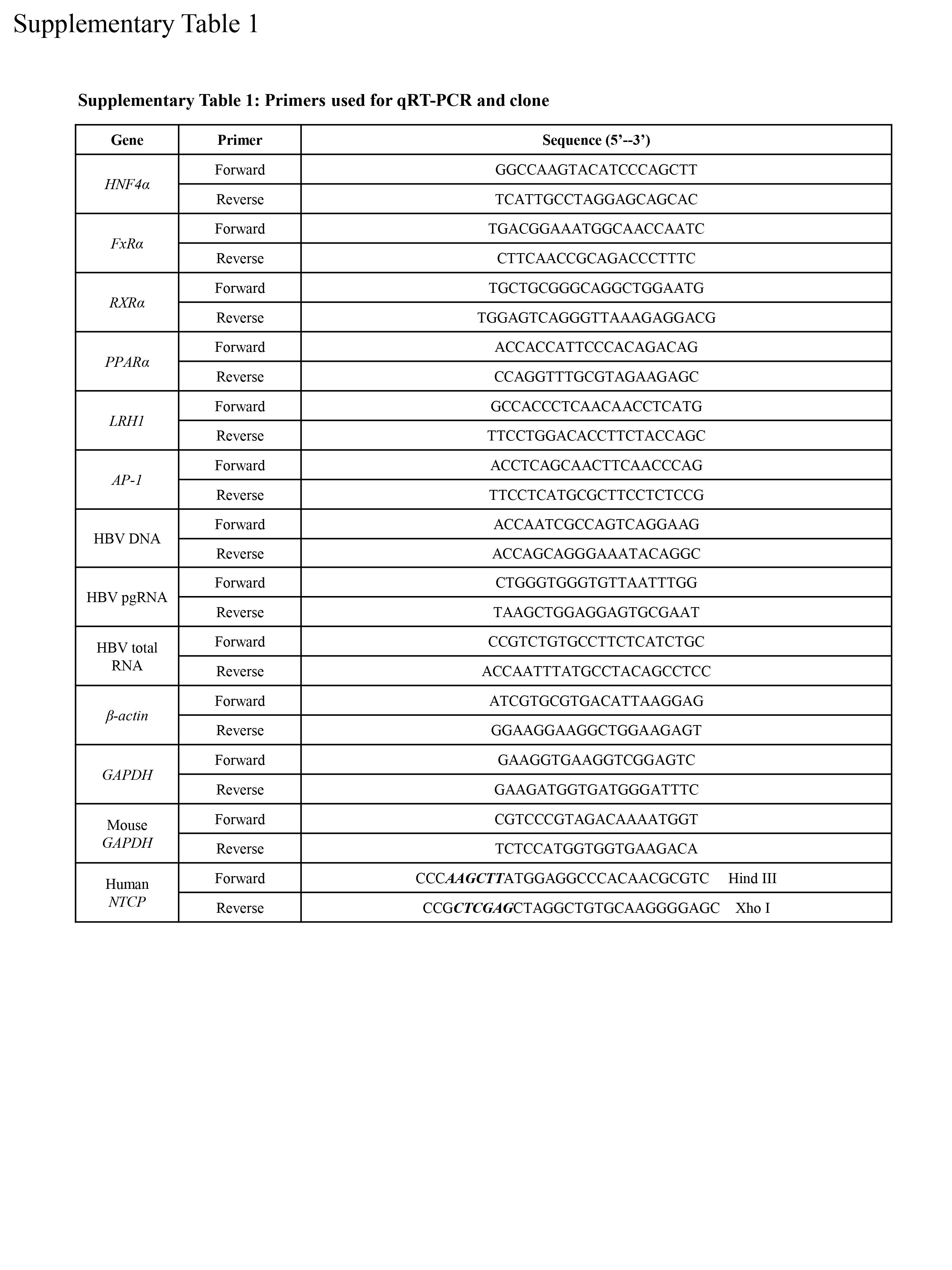

Supplement: Supplementary file 10 — Supplementary Table 1 [file 41426_2018_189_MOESM10_ESM.jpg]

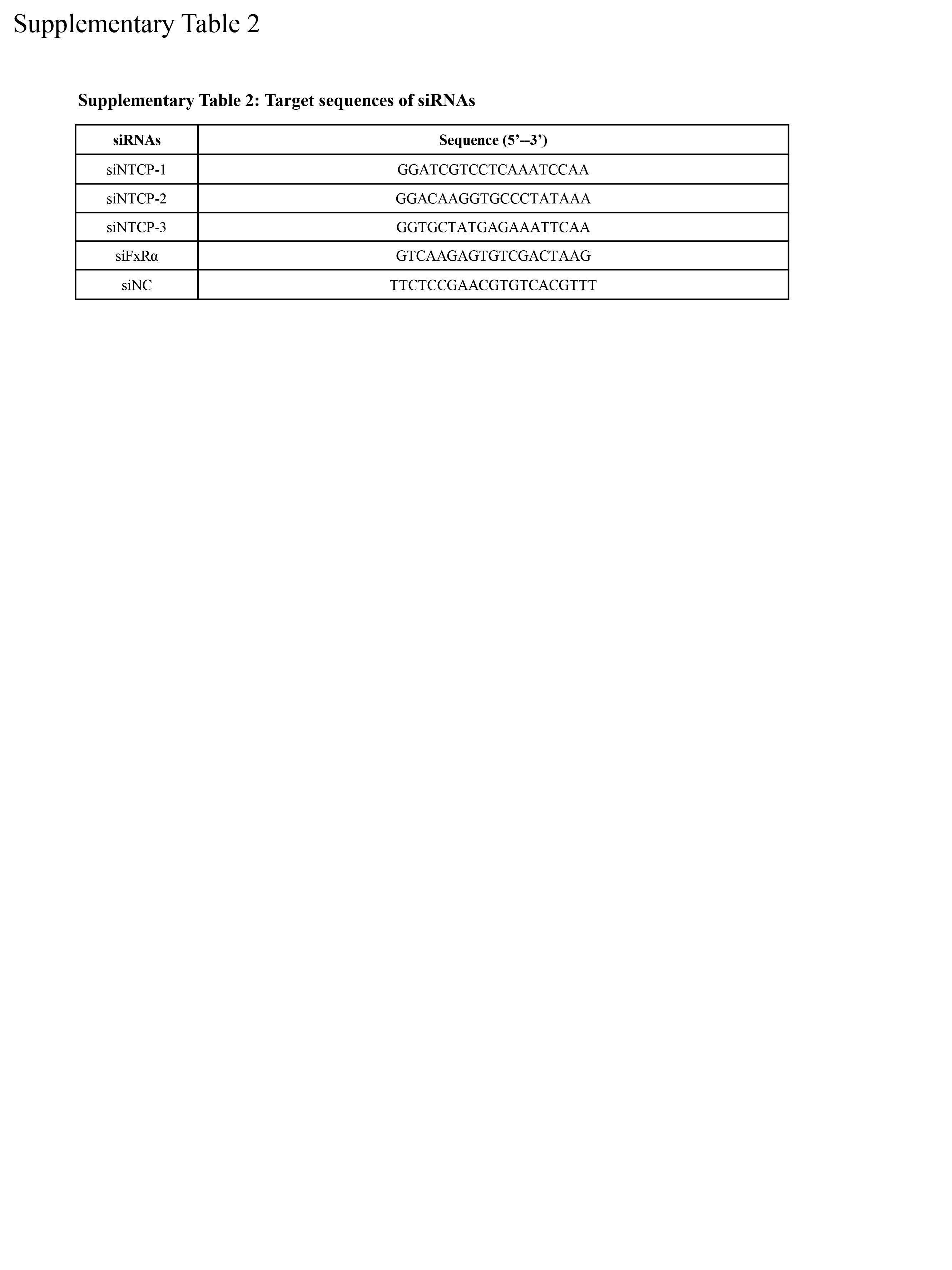

Supplement: Supplementary file 11 — Supplementary Table 2 [file 41426_2018_189_MOESM11_ESM.jpg]

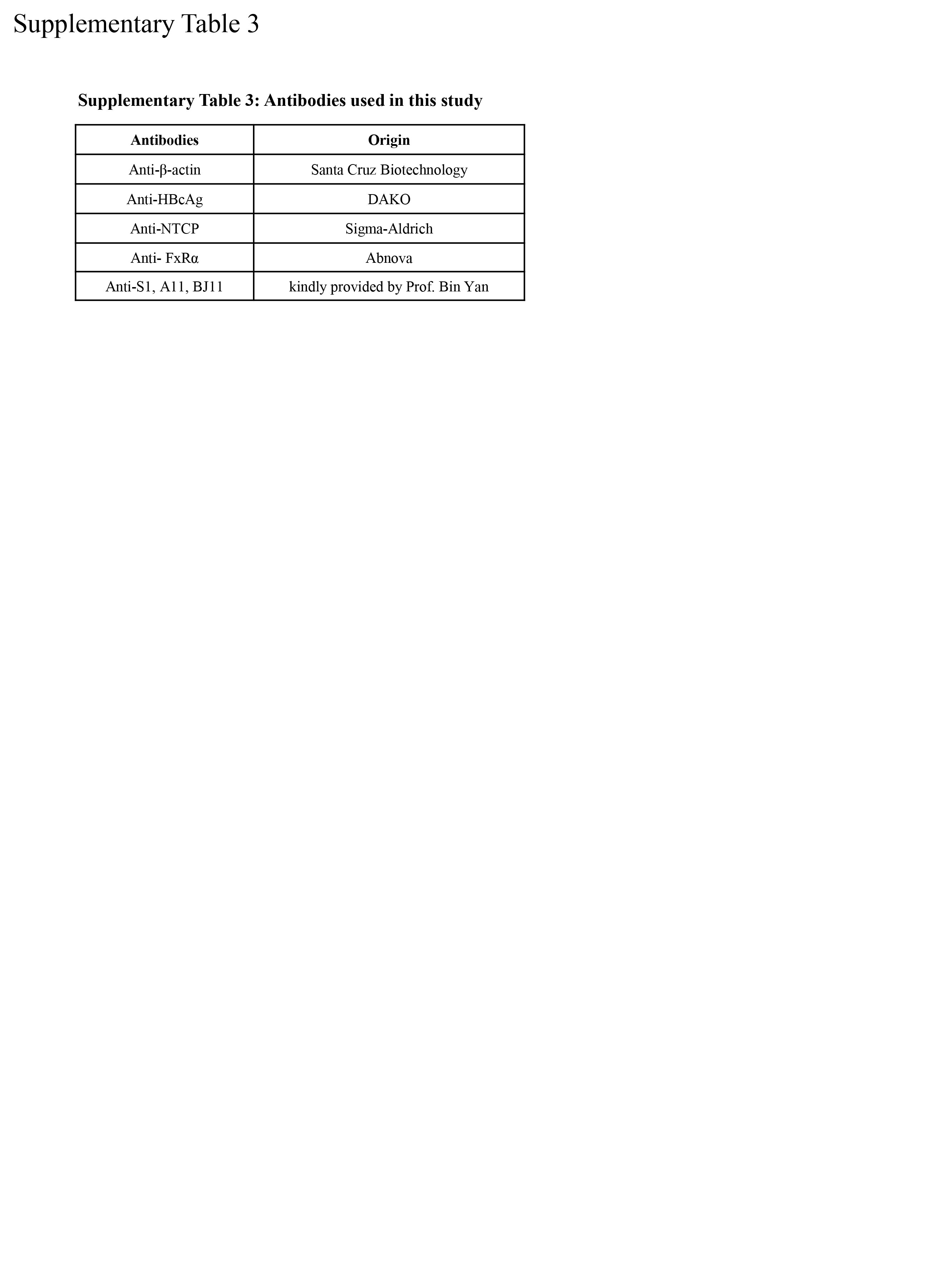

Supplement: Supplementary file 12 — Supplementary Table 3 [file 41426_2018_189_MOESM12_ESM.jpg]
